# Identification and validation of four hub genes involved in the plaque deterioration of atherosclerosis

**DOI:** 10.18632/aging.102200

**Published:** 2019-08-26

**Authors:** Peipei Chen, Yuexin Chen, Wei Wu, Lianfeng Chen, Xufei Yang, Shuyang Zhang

**Affiliations:** 1 Department of Cardiology, Peking Union Medical College Hospital, Chinese Academy of Medical Sciences and Peking Union Medical College, Beijing 100730, China; 2 Department of Vascular Surgery, Peking Union Medical College Hospital, Chinese Academy of Medical Sciences and Peking Union Medical College, Beijing 100730, China

**Keywords:** mRNA, robust rank aggregation approach, gene set enrichment analysis, atherosclerosis, biomarker

## Abstract

In recent years, intense research has been conducted to explore the diagnostic value of mRNA expression differences in atherosclerosis (AS). Nevertheless, because various technology platforms are applied and sample sizes are small, the results are inconsistent among the studies. We conducted a comprehensive analysis of a total of 161 tissue samples from 4 published studies after evaluating 230 datasets from the Gene Expression Omnibus and ArrayExpress. Adopting the newly published robust rank aggregation approach, combined with Kyoto Encyclopedia of Genes and Genomes pathway analysis, Gene Ontology functional enrichment analysis, and protein-protein interaction network construction, we identified four significantly upregulated genes (*CCL4*, *CCL18*, *MMP9* and *SPP1*) for diagnosing AS, even in the advanced stage. Then, we performed gene set enrichment analysis to identify the pathways that were most affected by altered mRNA expression in atherosclerotic plaques. We found that four hub genes cooperatively targeted lipid metabolism and inflammatory immune-related pathways and validated their high expression levels in ruptured plaques by qRT-PCR, western blot analysis and immunohistochemical staining. In summary, our study showed that these genes can be used as interventional targets for plaque progression, and the results suggested we should focus on small changes in these key indicators in the clinical setting.

## INTRODUCTION

Atherosclerosis (AS) is a chronic condition that has acute cardiovascular manifestations. Despite many advances in cardiovascular treatment and prevention, atherosclerotic cardiovascular disease and death are the leading causes of mortality and morbidity in developed and developing countries [1, 2], accounting for 31% of all deaths globally [3]. The rupture of atherosclerotic plaques as well as subsequent thromboses is the main cause of cardiovascular diseases such as stroke and heart attack [4]. Carotid plaque rupture leads to acute neurologic symptoms, and similarly, coronary plaque atheroembolism leads to acute coronary syndrome. The role of carotid endarterectomy in primary (asymptomatic patients) and secondary (symptomatic patients with nondisabling stroke or transient ischemic attack within the last 6 months) prevention of stroke in patients with severe carotid artery stenosis has been recognized [5]. However, one special challenge in combatting cardiovascular diseases is the abrupt and unforeseeable nature of the acute manifestations. Taking measures to prevent the destabilization and rupture of atherosclerotic plaques is the most appropriate therapeutic method for acute myocardial infarction. This strategy has fueled considerable research aimed at exploring new biomarkers to identify people who are at risk before any cardiovascular events occur to initiate primary prevention measures.

High-throughput technology has been utilized to identify differences in the expression levels of mRNAs between atherosclerotic plaques and normal tissues. These methods have the potential to recognize dozens or hundreds of differentially expressed mRNAs, although only a small portion of them might have real clinical utility as interventional targets. Finding an effective approach to combine various sources of data is important. Any difference in laboratory protocols or measurement platforms or small sample sizes could result in incomparable expression levels of genes. Therefore, researchers should analyze separate datasets and aggregate the resultant gene lists, as in the robust rank aggregation (RRA) [6] method. This approach has been used to define robust cancer-related gene sets [7] and miRNA sets [8–10]. However, this method has not been applied in AS research thus far.

In the RRA method, some individual research results are combined to increase the statistical power, and then, any discrepancy or inconsistency between different profiling studies is resolved. We adopted this comprehensive analysis method and then conducted pathway analysis to identify the physiological influence of the deregulation of mRNAs on the progression of AS. Furthermore, the potential hub genes were validated within the clinical setting by using quantitative real-time polymerase chain reaction (qRT-PCR). Finally, four promising mRNAs were selected. The aim was to find potential early-warning biomarkers and interventional targets to prevent atherosclerotic plaque destabilization and rupture after diagnosis in AS patients.

## RESULTS

### Robust rank aggregation (RRA) analysis of differentially expressed genes (DEGs) in differentiating plaque sets and poor prognosis sets from datasets

**Figure 1:**
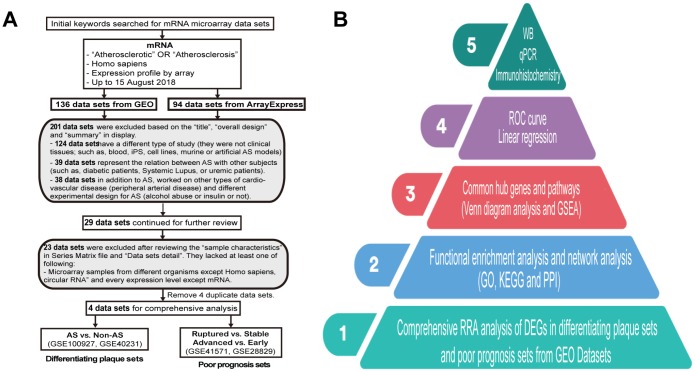
**Dataset selection flow chart and analysis processes.** (**A**) In total, 136 datasets from Gene Expression Omnibus (GEO) and 94 datasets from ArrayExpress were evaluated. Finally, 4 datasets for mRNAs were selected for inclusion in the comprehensive analysis. (**B**) The present study consisted of 5 steps, and the results of the analysis were finally validated on clinical samples.

During this research, we selected datasets of tissues from public databases so that we could compare and match them as much as possible. Hence, following the dataset selection in accordance with our criteria (Figure 1A), we analyzed four microarray datasets based on the workflow, and the hub genes were screened and verified in five steps (Figure 1B). After normalization and quality control, the four expression profiles for all datasets were analyzed using RRA analysis. A total of 161 samples were analyzed in our study, and their experimental design is shown in Table 1.

**Table 1:** Characteristics of the individual studies.

**GEO ID**	**Platform**	**Tissue type**	**Sample size**	**Citation (PMID)**	**Country**	**Time**
GSE100927	GPL17077 Agilent-039494 SurePrint G3 Human GE v2	Atherosclerotic carotid artery and healthy carotid arteries	29 vs.12	29500419 [48]	France	2018
GSE40231	GPL570 [HG-U133_Plus_2] Affymetrix Human	Atherosclerotic aortic wall and Internal mammary artery	40 vs.40	19997623 [49]	Sweden	2012
GSE41571	GPL570 [HG-U133_Plus_2] Affymetrix Human	Ruptured carotid atheromatous plaque and Stable carotid atheromatous plaque	5 vs.6	23122912 [50]	United Kingdom	2012
GSE28829	GPL570 [HG-U133_Plus_2] Affymetrix Human	Advanced carotid atherosclerotic plaque and early carotid atherosclerotic plaque	16 vs.13	22388324 [51]	Netherlands	2011

The GEO dataset and ArrayExpress are international public repositories in the context of high-throughput data. Abbreviation: GEO, Gene Expression Omnibus database.

**Figure 2:**
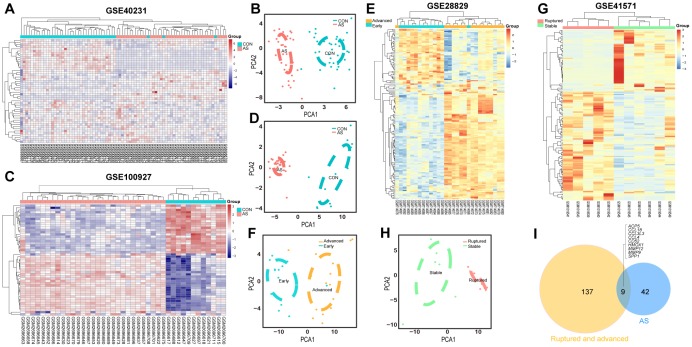
**Heatmaps and PCA score trajectory plots showing relative fold changes (FCs) of mRNAs in differentiating plaque sets and heatmaps and PCA score trajectory plots in poor prognosis sets.** (**A**, **C**, **E**, **G**) Heatmap showing 51 DEGs in the differentiating plaque sets and 146 DEGs in the poor prognosis sets after RRA analysis. In the differentiating plaque sets (GSE40231 and GSE100927), samples are sorted by columns, and genes are sorted by rows. Cyan squares represent the control group, and red squares represent the AS group. In the poor prognosis sets (GSE28829 and GSE41571), the blue/green square represents the early/stable stage of the AS group, and the yellow/red square represents the advanced/ruptured stage of the AS group. (**B**, **D**, **F**, **H**) PCA score trajectory plots showing obvious differences with those DEGs from RRA in the differentiating plaque sets or in the poor prognosis sets. (**I**) Venn diagram showing 51 DEGs in the differentiating plaque sets and 146 DEGs in the poor prognosis sets. A total of 9 shared hub genes were identified.

The GSE40231 and GSE100927 datasets, which served as the differentiating plaque sets, were shown to have 25 downregulated and 26 upregulated DEGs within AS plaques compared to control tissues after Bonferroni correction (Supplementary Table 5). Their heat maps showed that DEGs could discriminate the respective groups (Figure 2A, 2C), and the principal component analysis (PCA) score trajectory plots of AS did not substantially overlap with the profiles of the control group, indicating that the parallel PCA plots both showed apparent differences resulting from the AS state and control group (Figure 2B and 2D). Thus, these molecules were subjected to further analysis.

The expression profiles of poor prognosis sets were also analyzed; one had early or advanced carotid atherosclerotic plaques (GSE28829), and the other had stable carotid plaques compared with ruptured carotid plaques (GSE41571). After RRA analysis, 96 upregulated and 50 downregulated DEGs (Figure 2E, 2G and Supplementay Table 2) were identified. Their PCA plots also showed that the DEGs could clearly distinguish between the two groups in the poor prognosis sets (Figure 2F and 2H).

**Table 2:** Primers for real-time PCR.

**Gene**	**Forward (5′ to 3′)**	**Reverse (5′ to 3′)**
*GAPDH*	GGTGAAGGTCGGAGTCAACGGATTTGGTCG	GGATCTCGCTCCTGGAAGATGGTGATGGG
*hMMP9*	ATTTCTGCCAGGACCGCTTCTACT	CAGTTTGTATCCGGCAAACTGGCT
*hCCL4*	CTGTGCTGATCCCAGTGAATC	TCAGTTCAGTTCCAGGTCATACA
*hCCL18*	GGGGGCTGGTTTCAGAATA	CTCCTTGTCCTCGTCTGCAC
*hSPP1*	CTCCATTGACTCGAACGACTC	CAGGTCTGCGAAACTTCTTAGAT

### PPI network identification of potential hub genes in ruptured and advanced AS for functional enrichment analysis

**Figure 3:**
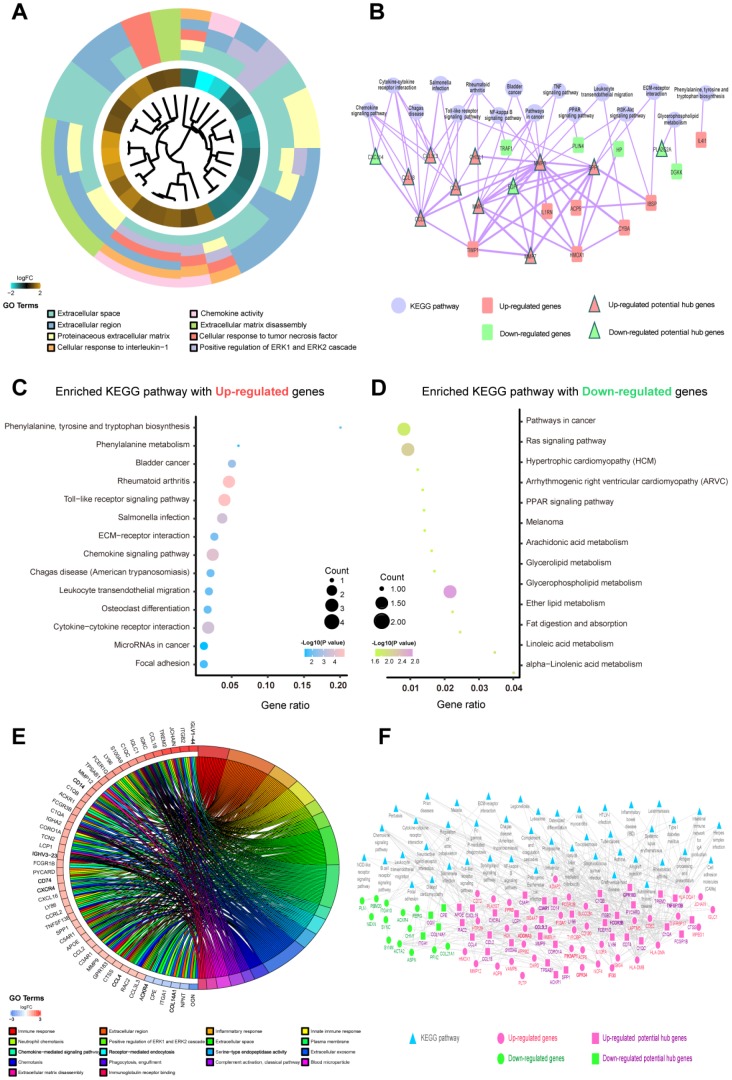
**Functional enrichment analysis of candidate genes in differentiating plaque sets and poor prognosis sets.** The potential hub genes were chosen using the p values corrected with the Holm step-down Bonferroni procedure. (**A**) GOCluster plot showing the relationship between 51 DEGs that were highly related to the AS state from RRA analysis and their related GO terms in differentiating plaque sets. For all genes, their high (low) logFC values are demonstrated by brown (turquoise) rectangles. (**B**) KEGG enrichment pathways and PPI network of the 51 DEGs were highly related to the AS state from the RRA analysis in differentiating plaque sets. The purple round node represents enriched pathways. Red rectangle nodes are upregulated genes, and red triangle nodes are upregulated potential hub genes. Green rectangle nodes are downregulated genes, and green triangle nodes are downregulated potential hub genes. The width of the line is proportional to the combined score of PPI. (**C**, **D**) An advanced bubble chart demonstrates enrichment of DEGs in signaling pathways in differentiating plaque sets. The Y-axis label is the pathway, and the X-axis label is the gene ratio (gene ratio=number of DEGs enriched in the pathway/amount number of all genes in background gene set). The size and color of the bubble represent the number of enriched DEGs of poor prognosis sets in the pathway and the significance of enrichment, respectively. (**E**) GOChord plot showing the 48 genes involved in more than 3 pathways and associated with ruptured and advanced plaques. Their contributions to the enrichment are arranged in the order of their level of expression. (**F**) KEGG enrichment pathways and PPI network of the 146 DEGs that were highly related to ruptured and advanced stages from the RRA analysis in poor prognosis sets. Blue triangular node: enriched pathways. Pink round node: upregulated genes, pink rectangular node: upregulated hub genes. Green round node: downregulated genes, green rectangular node: downregulated hub genes. The width of the line is proportional to the combined score of PPI.

A total of 9 shared hub genes were identified by Venn diagram (Figure 2I); these genes were identified by ranking and trend consistency based on the DEGs after RRA analysis, but relying on this analysis alone is one-sided. Pathway aggregation is often used to predict importance, and thus, we further used Gene Ontology (GO), Kyoto Encyclopedia of Genes and Genomes (KEGG) and protein-level protein-protein interaction (PPI) analyses to identify the 51 DEGs of the differentiating plaque sets and 146 DEGs of the poor prognosis sets. In the differentiating plaque sets, a GOCluster plot (Figure 3A) visualized the interaction between clusters and genes of GO terms, and the biological processes of the 51 potential hub genes were shown to focus on cellular response towards tumor necrosis factor, ERK2 and ERK1 cascade–positive regulation and cellular response to interleukin-1. Under molecular function, the genes were enriched in chemokine activity, and the analysis of the cellular components suggested that the genes were markedly enriched within the extracellular region, proteinaceous extracellular matrix and extracellular space (Figure 3A and Supplementary Table 1). In the KEGG pathway analysis, upregulated genes of the differentiating plaque sets were greatly enriched in 14 significant pathways, including cytokine-cytokine receptor interaction, chemokine signaling pathway and Toll-like receptor signaling pathway (Figure 3C). The downregulated genes were involved in 13 pathways, including glycerophospholipid metabolism, fat digestion and absorption, and glycerolipid metabolism, which were highly significant (Figure 3D). Figure 3C and 3D and Supplementary Table 2 show the detailed numbers of involved genes and these pathways in individual bubbles. Then, using CytoNCA analysis of the results in the PPI network, we identified 31 nodes (i.e., key genes in PPI network analysis) with statistical significance from 51 potential hub genes of the original differentiating plaque sets based on three network parameters, including closeness centrality, betweenness centrality and degree centrality of the constructed network. Next, the genes obtained by the above analysis (PPI network, GO analysis and KEGG analysis) in the differentiated plaque sets and their relationship between the related pathways and their gene expression changes were collectively plotted in Figure 3B. These key potential hub genes will be highlighted and will be explored and validated in the poor prognosis sets.

According to the method described in the analysis of differentiating plaque sets, the GOChord plot showed the functional enrichment results of the 146 potential hub genes in the poor prognosis sets, 48 of which were involved in more than 3 pathways and were associated with ruptured and advanced plaques (Figure 3E). These genes were involved in a total of 18 biological processes, such as immune, inflammatory, innate immune response, ERK2 and ERK1 cascade positive regulation, and other related pathways (Figure 3E and Supplementary Table 1). These data could help to further explore the functions of potential hub genes in the development and progression of atherosclerotic plaques. We also found a total of 45 KEGG pathways with significantly abundant DEGs (adj. *p*<0.05, Supplementary Table 3) in the poor prognosis sets. CytoNCA analysis was used to develop Figure 3F, which plots the interactive networks containing 81 nodes (i.e., key genes in PPI network analysis). We also obtained their key potential hub nodes by combining the above results of GO, KEGG and PPI in the poor prognosis sets, just as in the method of distinguishing plaque sets.

### Venn diagram identification of the common differentially expressed genes and pathways of the differentiating plaque sets and poor prognosis sets

**Figure 4:**
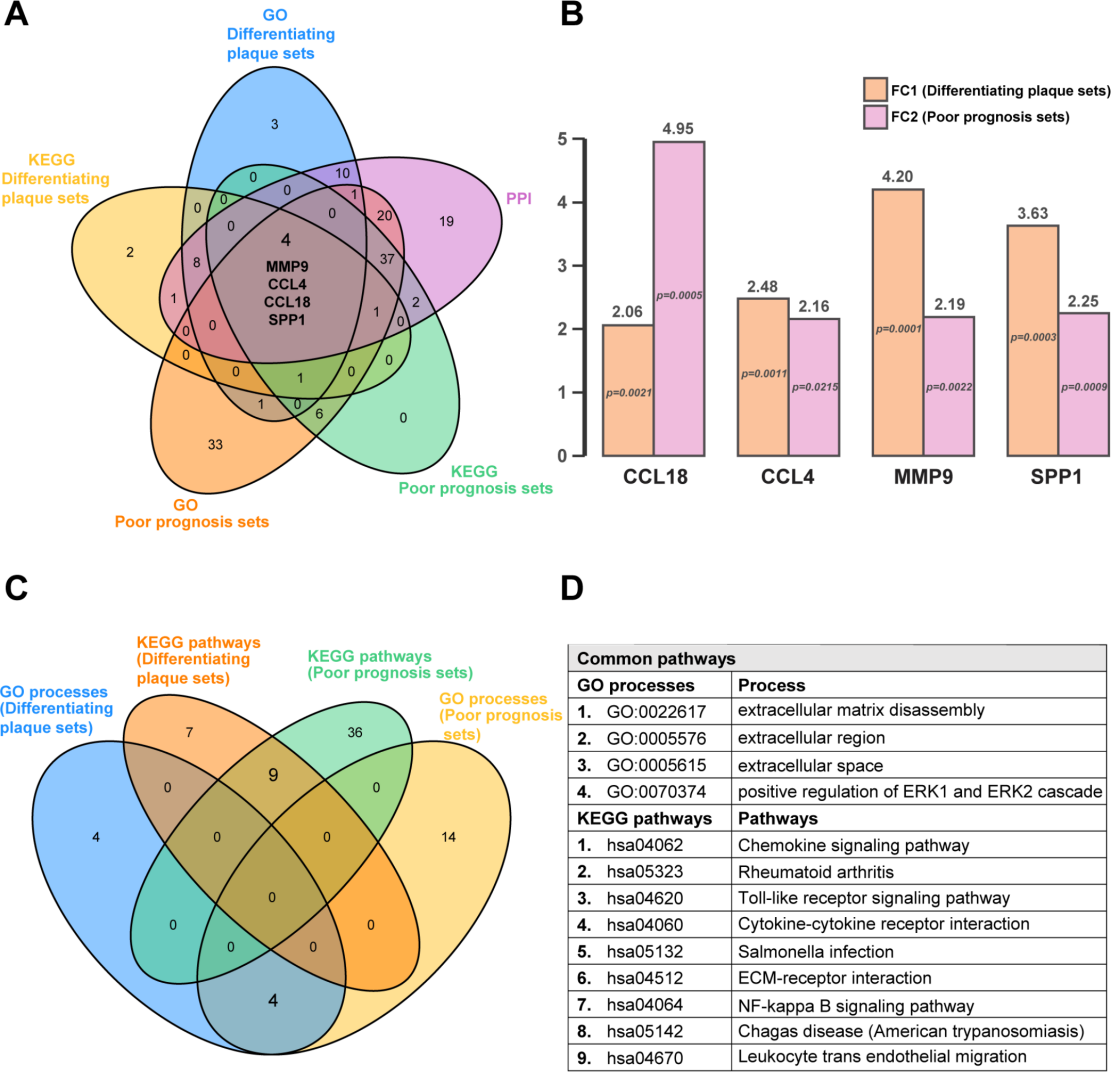
**Common hub genes and pathways in differentiating plaque sets and poor prognosis sets.** A five-set Venn diagram showing a combination of all differentially expressed genes of GO processes and KEGG pathways in differentiating plaque sets and poor prognosis sets. A total of 4 common genes were identified. Fold change and P value of the 4 common genes in differentiating plaque sets and poor prognosis sets. They are all upregulated genes. Four-way Venn diagram of GO processes and KEGG pathways identified in differentiating plaque sets and poor prognosis sets. A total of 4 GO processes and 9 KEGG pathways were identified in common between the training set and differentiating plaque sets. Details of the common pathways from Venn diagram analysis. FC_1_: fold change in differentiating plaque sets; FC_2_: fold change in poor prognosis sets.

Next, we used diagram analysis to select the genes and pathways of the differentiating plaque sets and poor prognosis sets, which were vital to distinguish the AS state and identify advanced-stage and ruptured plaques. The results identified the C-C motif chemokine ligand 18 (*CCL18*) gene, C-C motif chemokine ligand 4 (*CCL4*) gene, matrix metallopeptidase 9 (*MMP9*) gene and secreted phosphoprotein 1 (*SPP1*) (Figure 4A), which were all included in the 9 shared hub genes from RRA analysis (Figure 2I), indicating that they may be the best predictors or interventional targets for subsequent validation. They were all upregulated in both the differentiating plaque sets and poor prognosis sets, and the range of the fold change was 2.06 to 4.95 times (Figure 4B). In addition, 4 GO processes (including positive regulation of ERK1 and ERK2 cascades) and 9 KEGG pathways were common pathways to these two sets (Figure 4C and 4D). The Venn diagram analysis helped us identify common hub genes and pathways of the two sets as well as select important pathways and candidate genes that could be correlated with AS pathogenesis.

### ROC curve and linear regression analyses of the four common hub genes

**Figure 5:**
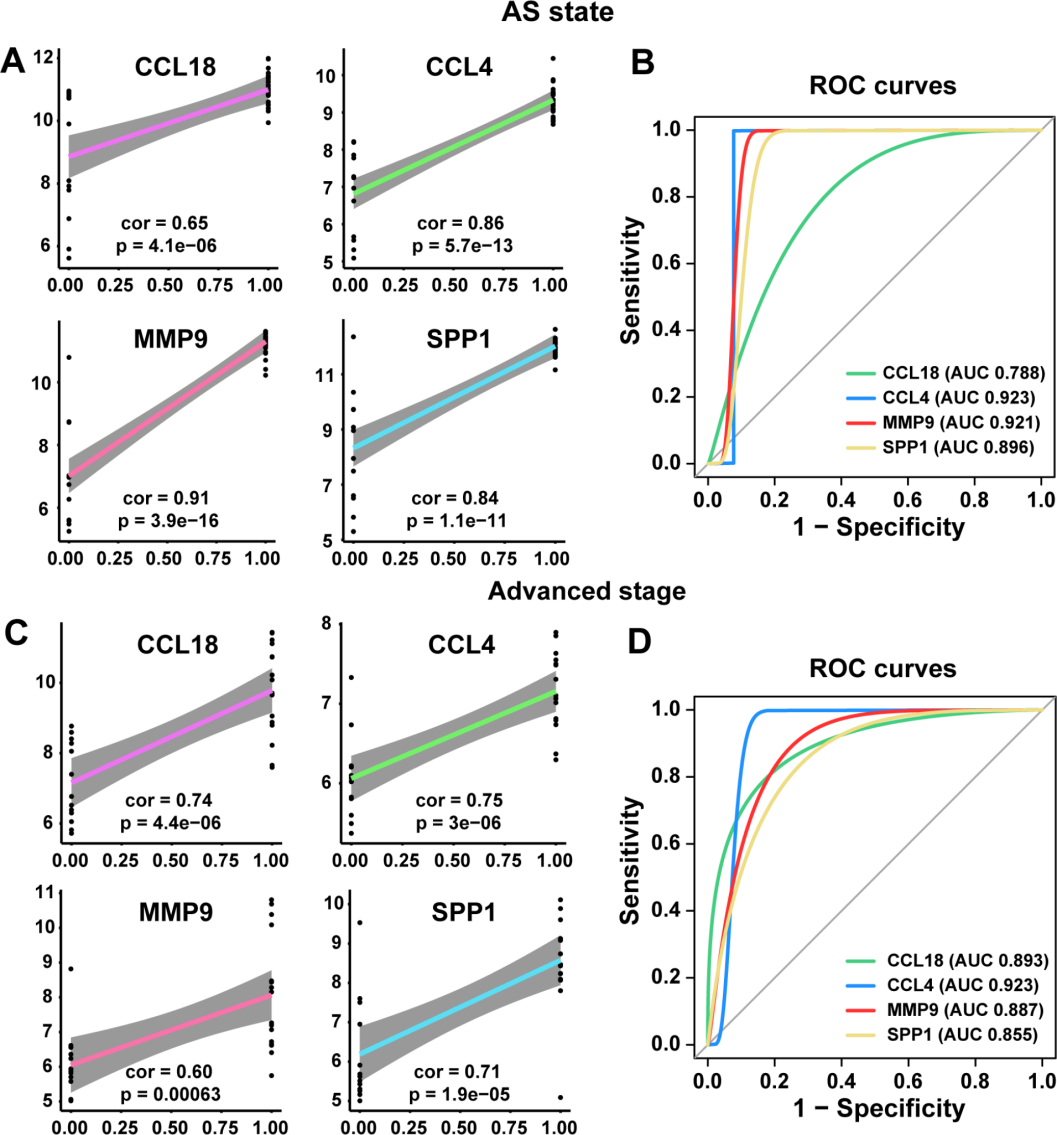
**Linear regression analyses and ROC curve in training and poor prognosis sets.** (**A**, **C**) The correlations of the expression of the 4 hub genes with AS state and advanced stage by linear regression analysis. (**B**, **D**) ROC curves of the 4 common hub genes for diagnosing the AS state or the advanced stage. According to one arbitrary guideline [31], we distinguished among excellent accuracy (0.9 ≤ AUC < 1), good accuracy (0.8 ≤ AUC < 0.9) and noninformative accuracy (AUC = 0.5).

Therefore, our next step was to conduct ROC curve and linear regression analyses of these common hub genes. The linear regression analyses indicated that the four common hub genes (*CCL18*, *CCL4*, *MMP9*, and *SPP1*) were positively correlated with the AS state and the advanced stage (all *p*<0.005, Figure 5A and 5C). Their ROC curves confirmed that they could distinguish the AS state and the advanced stage (all *p*<0.05), and the AUCs were between 0.788 and 0.923 (Figure 5B and 5D).

### Lipid metabolism and inflammatory immune-related pathways are progressively prominent in advanced-stage atherosclerotic plaques: Gene set enrichment analysis (GSEA)

**Figure 6:**
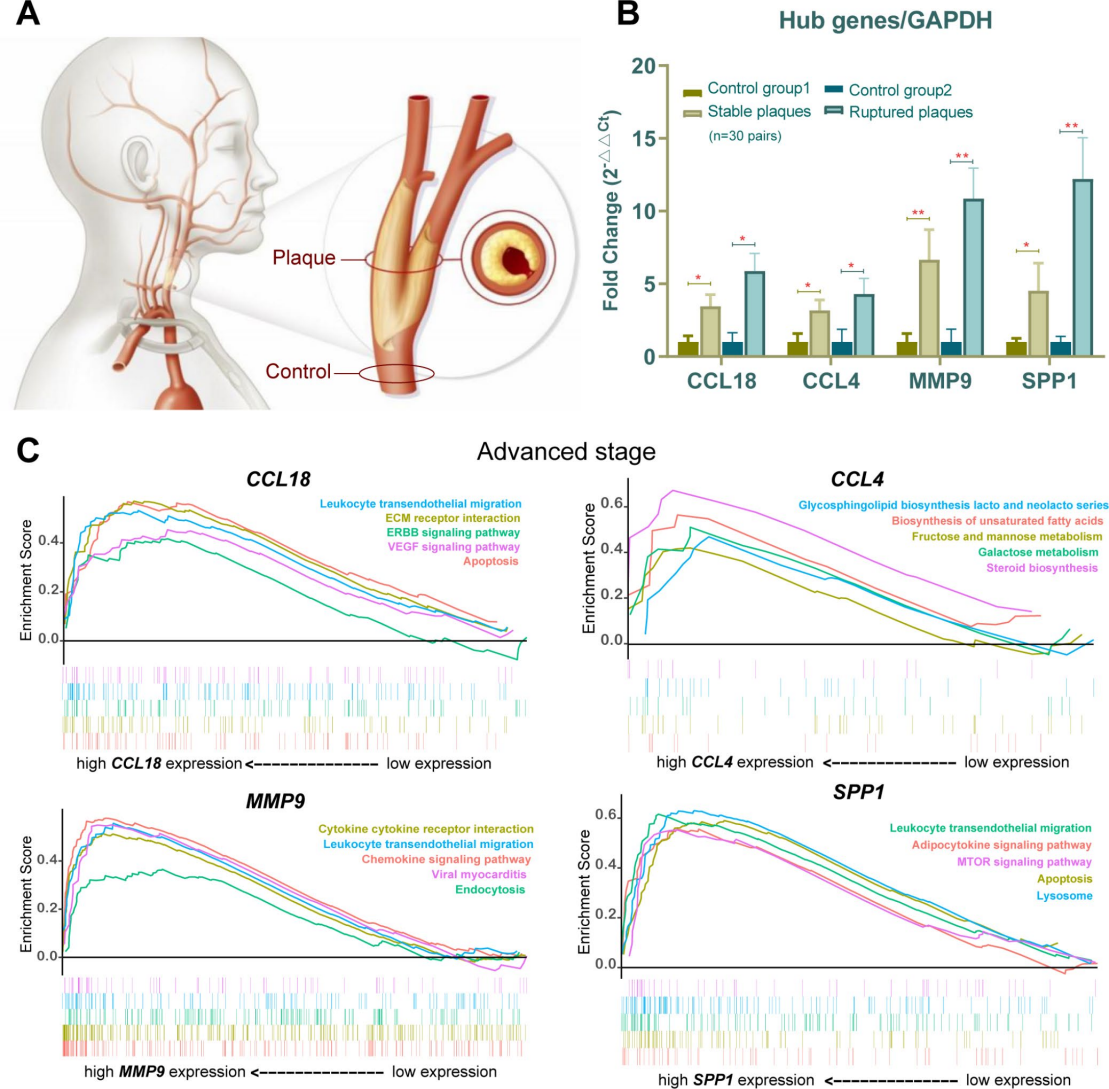
**Verification in the clinical samples and gene set enrichment analysis (GSEA).** (**A**) Human carotid artery segments were collected from below (normal control) and at (plaque-containing) the carotid bifurcation. (**B**) *CCL18*, *CCL4*, *MMP9* and *SPP1* expression in stable plaques (n=15 pair) and ruptured plaques (n=15 pair) were evaluated by qPCR and normalized against the corresponding glyceraldehyde-3-phosphate dehydrogenase (GAPDH) expression. An asterisk represents *p*<0.05, and two asterisks are shown as *p*<0.01 when compared with the normal control group. (**C**) Gene set enrichment analysis (GSEA) plots showing lipid metabolism and inflammatory immune-related gene sets progressively affected advanced-stage AS.

To determine the possible functional pathways of these hub genes at the advanced stage of atherosclerotic plaques without ignoring their slight changes at the beginning of the late stage (their expression changes had not yet reached a significant level), we performed a GSEA to map the biological processes of these genes (Figure 6C).

**Figure 7:**
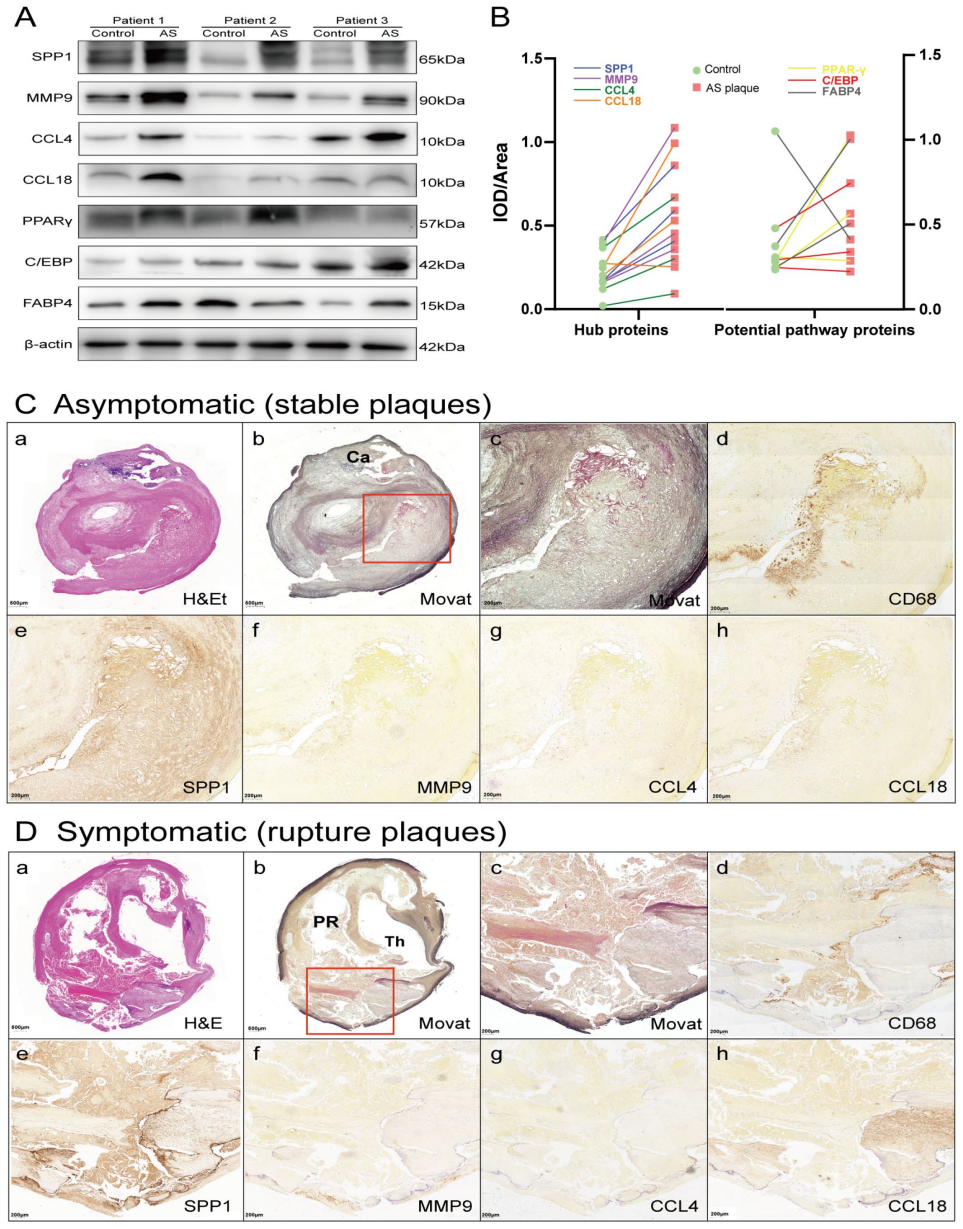
**Hub proteins and potential pathway proteins were increased in AS plaques, especially in tissue sections of ruptured plaques.** (**A**) Detection of hub proteins (SPP1, MMP9, CCL4, and CCL18) and potential pathway proteins (PPARγ, C/EBP and FABP4) in ruptured plaques and adjacent normal tissues by western blots. β-actin was used as a loading control. Bands were quantified with ImageJ software. (**B**) Line chart showing IOD/area of proteins in immunoblot analysis. Red squares represent AS plaques, and the green circles indicate adjacent normal tissues. The different colored lines represent the trend of protein expression. (**C**, **D**) H&E (a) and Movat (b and c, low and high power) staining and immunoperoxidase antibody staining using anti-CD68 (d), anti-SPP1 (e), anti-MMP9 (f), anti-CCL4 (g), and anti-CCL18 (h). Ca, calcification; PR, plaque rupture; Th, thrombus.

GSEA is particularly useful for identifying the correlations between related pathways of genes. The results revealed that the expression of the 4 hub genes was positively correlated with lipid metabolism and inflammation-related pathways. For example, lipid metabolism–related gene sets for galactose metabolism, adipocytokine signaling pathway, glycerolipid metabolism, and steroid biosynthesis pathways were upregulated at the advanced stage, with high enrichment on the basis of increasing gene expression, as well as the inflammatory immune-related lysosome, leukocyte transendothelial migration, apoptosis and chemokine signaling pathway (Figure 6C). Expression levels of peroxisome proliferator–activated receptor γ (PPARγ), C/EBP and fatty acid binding protein (FABP4), which are important markers of adipogenesis and adipocytokine pathways, were increased in AS plaques compared with the control samples (Figure 7A, 7B). These results suggested that slight changes in the expression of the 4 hub genes might also cause lipid metabolism and inflammatory immune-related pathways to become progressively enhanced in advanced-stage atherosclerotic plaques, suggesting that researchers should focus on small changes in key indicators in the clinical setting.

### Verification in the clinical samples: qRT-PCR, western blotting and immunohistochemistry

To confirm and validate the expression of the 4 common hub genes that were determined from the microarray data analysis, we included clinical samples of 30 patients suffering from carotid artery stenosis in total (>70%, determined by ultrasonography) in the present study. The symptomatic patients with ruptured plaques showing neurological symptoms (nondisabling stroke or transient ischemic attack within the last 6 months) were assigned from the Department of Vascular Surgery of Peking Union Medical College Hospital, Beijing.

The mRNA expression of the 4 common hub genes (*CCL18*, *CCL4*, *MMP9* and *SPP1*) was examined using qRT-PCR in 30 pairs clinical samples (15 stable plaques vs. control group 1 and 15 paired ruptured plaques vs. control group 2, n=30 pairs). The results from qRT-PCR showed that the mRNA levels of *CCL18*, *CCL4*, *MMP9* and *SPP1* increased by 3.46-, 3.18-, 6.67- and 4.52-fold in stable plaques compared with normal controls, respectively (Figure 6B). The above genes were more prominently elevated in the ruptured plaques, and the mRNA levels of *CCL18*, *CCL4*, *MMP9* and *SPP1* were increased by 5.88-, 4.31-, 10.86- and 12.21-fold compared with those of the adjacent normal controls. Western blotting results showed that the expression levels of these hub proteins (SPP1, MMP9, CCL4, and CCL18) increased in ruptured plaques compared with adjacent normal tissues (Figure 7A, 7B). The results are consistent with the data collected from our comprehensive bioinformatics analysis above. We also verified the adipogenesis and adipocytokine pathway proteins (PPARγ, C/EBP and FABP4) in ruptured plaques, which showed mild to moderate elevations (Figure 7A, 7B).

Ten carotid histologic sections were evaluated. The most frequent plaque morphology was fibroatheroma in both symptomatic and asymptomatic plaques. Plaque rupture was more commonly observed in symptomatic plaques than asymptomatic plaques. Fibrocalcific plaques and/or calcified nodules were more common in asymptomatic plaques than symptomatic plaques. Representative histologic images from stable and ruptured plaques are shown in Figure 7C. The necrotic core area was significantly greater and the extent of calcification was significantly lower in ruptured plaques compared with stable plaques. Immunohistochemical analysis revealed that ruptured plaques had a greater area occupied by CD68 macrophages and significantly greater expression of SPP1 and CCL18 than plaques from asymptomatic patients. There was a trend towards increased expression of MMP9 and CCL4 in ruptured plaques, although the differences compared to stable plaque lesions were not significant.

## DISCUSSION

In the present study, we first identified 51 mRNAs in AS based on two mRNA profiling studies by using the RRA method. Twenty-six upregulated mRNAs and twenty-five downregulated mRNAs had a good diagnostic ability (Supplementary Table 5). Next, we investigated two other independent public databases, including 40 tissues from advanced and ruptured stages. The bioinformatics analysis suggested that the high expression of *CCL18*, *CCL4*, *MMP9* and *SPP1* had good value in differentiating plaques and even identifying advanced and ruptured stages. The results of ROC curve and linear regression analyses validated these results. Then, we further obtained the expression differences in these 4 genes between our matching carotid atherosclerotic plaque samples in advanced and ruptured stages and controls (n=30 pairs) by using qRT-PCR, western blotting and immunohistochemistry, which were consistent with the above bioinformatics results. As expected, our comprehensive analysis identified the strong clinical value of these genes. Additionally, GSEA revealed that slight changes in the expression of these hub genes within atherosclerotic plaques were positively correlated with lipid metabolism and inflammation-related pathways. Therefore, we should focus on small changes to key indicators in the clinical setting. Taken together, our data provide evidence for the four genes as tissue-specific marker cues and can be served as indicators of future interventions.

Datasets of mRNA expression profiling lack consistent results between studies because of the application of different laboratory protocols and technology platforms and small sample sizes. Although the optimal approach is to pool them together, such a strict method is usually unfeasible because of the different platforms. To overcome this limitation, researchers could analyze separate datasets and aggregate the resultant gene lists. Here, we adopted the RRA approach [8,11,12] to analyze mRNAs in advanced and ruptured AS obtained from independent profiling experiments. The core element of this method is the search for the most commonly recognized genes among different studies.

Usually, self-organizing map analysis and individual gene-based analysis can identify genes with significant expression changes. Nevertheless, using these two methods may miss the subtle differences in genetic expression of functionally and biologically related gene sets in response to AS status or progression stage. To overcome the shortcomings of this analysis, we used the popular GSEA method [13, 14] to conduct a comparative study of different gene set enrichment methods for the four hub genes between two groups (poorly expressed or highly expressed in the advanced AS group). GSEA is more powerful than traditional single-gene approaches for exploring the effect of gradual change in expression of target genes in a specific disease stage, such as the advanced AS stage in this study (Figure 6C).

We performed a comprehensive analysis of four mRNA profiling databases by evaluating 136 datasets from GEO and 94 datasets from ArrayExpress. To our knowledge, this is the first comprehensive research that combines all data on mRNA research at the tissue level from the public database and specifically investigated the common hub genes of the human AS state and of the advanced and ruptured stages of AS. This study proposed four promising mRNAs that could provide some clues to future intervention targets and their underlying mechanisms. These data of our study will also help predict the clinical deterioration of patients with AS plaques in the advanced and ruptured stages.

When using mRNAs as candidate prognostic and diagnostic biomarkers for AS, some factors should be taken into account. First, a biomarker’s fold change is supposed to be significant enough to differentiate AS tissues from control tissues and even discriminate between the advanced and ruptured stages. The average fold changes of the four identified upregulated common hub mRNAs (*CCL18*, *CCL4*, *SPP1*, *MMP9*) from datasets (Figure 4B) or in our human carotid plaques validated by qRT-PCR (Figure 6B) were all more than twice, and their expression levels also increased in the AS plaques, as shown by immunoblot analysis. The expression levels of CCL4 and CCL18 were not obvious in asymptomatic stable plaques, which are easily lost during immunohistochemical manipulation because they are secreted proteins; however, they were shown to be significantly increased in AS plaques by the more sensitive mRNA and protein expression experiments.

Second, the biological function of all mRNAs should be investigated comprehensively so that we can use them in clinical settings. The GSEA of putative target genes indicated that variation of the expression of the 4 hub genes may influence the lipid metabolism and inflammatory immune-related pathways involved in AS progression (Figure 6C). In our GSEA list, steroid biosynthesis, biosynthesis of unsaturated fatty acids, cytokine-cytokine receptor interaction, lysosome, chemokine signaling pathway and leukocyte transendothelial migration were ranked at the top; these findings are consistent with the known primary functions of these hub mRNAs. Then, the markers of the lipid metabolism synthesis pathway were further explored by immunoblotting. Activated nuclear receptor PPARγ is a master regulator of adipogenesis, acting as a transcription factor of FABP4 expressed in mature adipocytes [15]. A previous study [16] demonstrated that the differentiated state of adipose cells is achieved and maintained via a cycle of positive cross-regulation between C/EBPα and PPAR-γ. PPAR-γ, C/EBP and FABP4 are important markers of adipogenesis and adipocytokine pathways, consistent with the four hub protein trends and increased in ruptured plaques (Figure 7A, 7B).

These four hub genes have been shown to be matrix-related factors (MMP9 and SPP1) or inflammatory factors (CCL18 and CCL4), which are increasingly secreted when cell integrity is disrupted or inflammatory stimuli occur. For example, the chemokine CCL18 serves as a marker of anti-inflammatory activation and has been validated as a specific marker of refractory unstable angina pectoris [17]. CCL18 might participate in human atherosclerotic plaque formation [18]. LPS-induced *CCL4* production in human monocytes has a significant positive correlation with LDL and total cholesterol concentration in vitro [19], and increasing expression of *CCL4* in peripheral blood of patients with coronary artery disease was reduced by statin therapy [20]. The above pathway is consistent with the GSEA finding of this study that abnormalities of *CCL4* may affect lipid metabolism gene sets (Figure 6C). MMP-9 modulates cholesterol metabolism, which affects the hepatic transcriptional responses to dietary cholesterol [21], and MMP-9 is associated with dysfunctional HDL and its proinflammatory properties [22]. Thus, dysregulation of *MMP-9* can result in metabolic disorders, which could promote the formation of AS. In vitro, MMP-9, which is mainly derived from macrophages, plays dual roles in AS regulation, and MMP-9 cleaves extracellular matrix (ECM) substrates (particularly collagen) within the fibrous cap to increase the vulnerability of the plaque and promotes the migration of smooth muscle cells (SMCs) as well as transforming growth factor (TGF)-β1 signaling to enhance ECM deposition [23]. However, the contribution of all effects to on the development of plaque remains to be elucidated, and the effects of the 4 hub genes on each other have not been confirmed. SPP1 is also known as osteopontin and is an adhesion molecule and proinflammatory cytokine implicated in monocyte chemoattraction and cell-mediated immunity. Previous studies [24, 25] indicated that genetic *SPP1* deficiency weakens AS development within apoE^-/-^ mice, and macrophage *SPP1* expression regulation is one mechanism whereby LXR ligands might influence the AS and inflammatory responses of macrophages. *SPP1* mRNA has been observed within the wall near atheromas and is closely related to calcification [26]. When both SPP1 transgenic and wild-type mice were exposed to atherogenic diets, the former exhibited enhanced aortic atherosclerotic disease. Furthermore, foamy macrophages within their atherosclerotic plaques expressed higher levels of SPP1 than such macrophages in control mice [27]. In ApoE–/– mice exposed to AngII, SPP1 deficiency not only caused a reduction in the size of the aortic atherosclerotic lesions but also decreased the abundance of macrophages as well as their viability in the atherosclerotic plaque [28]. Thus, SPP1 contributes not only to plaque formation but also to plaque instability. During angiogenesis and vascular remodeling, behaviors of vascular smooth muscle cells (VSMCs) and their interaction with ECM play a critical role in the processes. Rat VSMCs overexpressing MMP9 showed enhanced migration and invasion in a collagen invasion assay [29]. Genetic MMP9 knockout impaired the migratory activity of isolated VSMCs and decreased intimal hyperplasia [29, 30]. In addition, a lack of MMP9 caused reorganization of the collagenous matrix and reduced VSMC attachment to gelatin [30, 31]. Studies have also found that VSMC replication is significantly decreased in MMP9(−/−) arteries and that MMP9 may regulate VSMC proliferation by modulating cell adhesion as well as the cadherin and β-catenin association [32]. These findings indicate that MMP9 not only degrades ECM but also maintains a connection between the cell surface and matrix. Various cytokines induced in vascular injury and immunoinflammatory responses contribute to AS and restenosis through MMP9-mediated VSMC migration. For CCL18, as mice appear to have fewer chemokine genes than humans, no CCL18 homologue has been found in rodents thus far [33]. RT-PCR analysis and in situ hybridization had demonstrated that CCL18 was only expressed in human atherosclerotic plaques and that the mRNA was restricted to macrophage-rich areas of the lesions [34]. The accumulation of macrophages in the arterial tunica intima plays an important role in the development of AS. CCL4 has not been extensively studied, similar to other members of the CC chemokine subfamily, and there are currently no studies of corresponding knockout mice. CCL4 was also identified upregulated in vulnerable AS plaques and was expressed by T cells in advanced atherosclerotic lesions in stroke patients, indicating that it might play a potential role in the development of AS [35, 36].

Third, there should be sufficient information on mRNA expression patterns within different specimen types. In the context of partial inconsistencies between plasma-based and tissue-based results [37], we focused on research that analyzed the expression of mRNAs between AS plaques and control tissues, further exploring if DEGs of the AS state were also in the plaques of advanced and ruptured stages, because we thought the results could be tissue-specific. Although some studies have reported that circulating mRNAs in plasma could be more convenient and timely to diagnose AS than other molecules, we focused on tissue-specific hub genes to provide targeted directions for subsequent drug interventions. Large sample data in previous studies confirmed that partly genes are elevated in the peripheral blood of patients with coronary heart disease or AS and indirectly demonstrated that they can also be monitored as noninvasive markers. Additionally, chemokines play important roles in the pathology of AS and related cardiovascular diseases. The human plaque samples of the previous studies came from fewer than 40 cases, and there may have been bias, so greater sample numbers would be more reliable and persuasive. The human plaque samples involved in the analysis in our study reached 120 cases (90 cases from the public database and 30 cases from clinical validation). Among the four mRNAs in this study, CCL18 was significantly upregulated in AS plaques. After strict filtering, the expression of *CCL18* and *SPP1* were still increased. The concentrations of SPP1 and CCL18 have been significantly and consistently upregulated within the local versus peripheral blood of acute myocardial infarction [38]. One review reported that high plasma SPP1 was related to the increased risk of major adverse cardiac events [39] and to the extent and presence of coronary artery disease [40]. Nevertheless, no studies have reported the function of SPP1 within carotid plaque tissues. In our study, all RNA levels and protein levels and the results of immunohistochemistry showed that SPP1 expression was obviously increased in ruptured carotid plaques. *CCL4* has also been detected in human AS plaques [41], and its plasma level reflects the level of proatherogenic cytokines in plaque tissue [42]. These data, combined with our findings, support *CCL4*’s potential role in atherosclerotic disease and plaque vulnerability. *MMP-9*, one of the four hub genes, is a critical enzyme released from macrophages. Elevated serum MMP-9 is independently associated with plaque instability [43] and severity [44]. Another study revealed that the role of MMP9 in the degradation of atherosclerotic fibrous caps leads to fissures and eventually to acute thrombosis [45] and might be a target for unstable plaque treatment [46]. Therefore, *MMP9* may be a tissue-specific potential diagnostic marker of the ruptured stage of AS. These data illustrated the reliability of our bioinformatics analysis. These findings provide a new idea for further exploration of the progression of AS.

Last but not least, it is necessary to rigorously validate and demonstrate reproducibility within one independent cohort of patients to confirm the prognostic and diagnostic value of these common hub genes. We validated the 4 candidate hub genes within AS samples through experiments and verified that their expression was consistent with the results of the bioinformatics analysis in this study. We used a comprehensive analysis that combined some individual research results to increase the statistical power and then resolved the inconsistency between various profiling studies. This method identified potential hub genes that were stable and critical for various functions. Thus, researchers should obtain a holistic view of candidate mRNAs from multiple studies to avoid one-sided opinions.

Nevertheless, we must admit that our analysis has certain limitations. The main limitation is the strict control of filtering conditions in the bioinformatics analysis of this study. For example, only DEGs with coexisting results of GO enrichment, KEGG signaling pathway analysis and the correlation score list of the PPI network could be considered potential hub genes. Therefore, the screened genes did not include all of the AS-related genes that have been reported thus far. Our conclusions will be more convincing if further prospective studies of multicenter clinical trials are performed. Third, the present study was validated with carotid plaques. AS is a disease affecting large and medium elastic and muscular arteries, especially in turbulent flow vessels, and the symptoms of coronary heart disease caused by coronary AS are the most urgent among AS. Considering the difficulty in obtaining coronary atherosclerotic plaques, we have combined the results of carotid plaques and bioinformatics analysis to explore the possibility of these four hub genes as intervention targets. Although previous studies have linked them to disease in the blood, in the future by designing a large sample cohort study could be useful in clinical practice. AS is complicated pathologically, and no single biomarker is optimal. These four biomarkers should be used together to monitor and diagnose the progression of AS or the efficacy of treatment.

Our study proposed an approach to resolving the differences between studies and may provide clinical value for research on mRNAs in AS. The 4 identified hub genes (*CCL18*, *CCL4*, *MMP9* and *SPP1*) might be utilized as diagnostic biomarkers or even prognostic factors, and they could serve as targets for interventions of plaque progression.

## MATERIALS AND METHODS

### Study design and data collection

Four microarray datasets (GSE40231, GSE100927, GSE28829 and GSE41571) of human AS were obtained from the NCBI Gene Expression Omnibus (GEO) and ArrayExpress in accordance with our criteria up to August 4, 2018 (Figure 1). The former two were used for gene expression profiles for AS, while the latter two were analyzed for ruptured and advanced stages of AS. A total of 161 samples (71 control or early stage and 90 cases or advanced stage) were analyzed in our study (Table 1). They were gathered from patients or donors in different countries. These four datasets were produced independently utilizing the GPL17077 and GPL570 platforms, and the normalization and quality control of these data were carried out with the “affy” R package [47] in our study.

### Differentially expressed gene (DEG) screening and principal component analysis (PCA) in testing and differentiating plaque sets

Data analysis was performed using the “limma” R package [52] to detect each DEG between the AS state and control or early stage and advanced stage after normalizations. The |log_2_fold change (FC)|>1 and false discovery rate (FDR)<0.05 were used as the cut-off criteria. Heatmaps and volcano plots were plotted using the pheatmap [53] and ggplot2 [54] package. Two features were extracted from the genes of each group using an unsupervised PCA method.

### Comprehensive analysis by the robust rank aggregation (RRA) method

Different expression profiles of genes might show DEGs to obtain precise gene expression levels [55]. Therefore, we conducted a comprehensive analysis utilizing the “RobustRankAggreg” R package [6]. This approach assigns a p value to all elements in the list aggregated, indicating how much better its rank is than that of the null model, which expects random ordering. For assessing the stability of the obtained p values, leave-one-out cross-validation was utilized within the RRA algorithm [56]. The analysis was repeated 10,000 times, and a random gene list was left out of the analysis each time. Then, the obtained p values from all rounds for all mRNAs were averaged. We conducted one subcomprehensive analysis of AS or non-AS samples (GSE40231 and GSE100927), and another analysis was performed in early- or advanced-stage and ruptured or stable plaques (GSE28829 and GSE41571) because they were different stages of AS, which served as the poor prognosis sets.

### Enriched Gene Ontology (GO) functional enrichment analysis, Kyoto Encyclopedia of Genes and Genomes (KEGG) pathway analysis and protein-protein interaction (PPI) network construction

To explore the potential mechanism of how DEGs in comprehensive analyses impact the correlative AS state and advanced stage, we uploaded potential hub genes from RRA analysis into the Database for Annotation, Visualization and Integrated Discovery (DAVID) [57] database and performed GO functional enrichment analysis [58] and visualization on KEGG pathway maps by KOBAS [59]. A false discovery rate (FDR)<0.05 was used as the cut-off criterion. We used GOChord plot functions of the GOplot R package to add quantitative information about molecules to the GO terms of interest [60], which permitted us to incorporate data obtained from the measurements of the expression level with the data derived from functional annotation enrichment analysis. Additionally, the CytoNCA plug-in [61, 62] was applied to Cytoscape 3.2.1 [63] using betweenness centrality (BC), degree centrality (DC), and closeness centrality of the nodes of the PPI network as evaluation. A confidence score greater than 0.4 in the PPI network was considered significant. In this part, the common genes in the above three analyses served as common hub genes.

### Venn diagram analysis

Venn diagram analysis was conducted by the VennDiagram R package [64, 65] for DEGs and pathways, which were filtered from datasets by GO, KEGG and PPI methods, and the results of RRA. The diagrams visualized the overlapping genes and biologically complementary aspects.

### Diagnostic effectiveness evaluation

For diagnostic analysis, the patients were divided into 2 groups in accordance with the expression of hub genes (low vs. high). Trait correlations were calculated and plotted using the corrplot package [66]. We investigated the optimal cut-off value by maximizing the Youden index, plotted the ROC curve, and calculated the AUC with the “ROCR” package [67]. We used one guideline [68] to distinguish between excellent accuracy (0.9≤AUC<1), good accuracy (0.8≤AUC<0.9) and noninformative accuracy (AUC=0.5). When the AUC value of the hub gene was greater than 0.8, it was considered to have excellent specificity and sensitivity to distinguish between the AS state or advanced stage.

### Ethics statement and sample collection

This study was approved by the Department of Cardiology and Department of Vascular Surgery of Peking Union Medical College Hospital, Chinese Academy of Medical Sciences and Peking Union Medical College (Beijing, China). Symptomatic patients (n=15, average age: 66.8 years, age range: 55–78 years) and asymptomatic patients (n=15, average age: 65.1 years, age range: 60–70 years) with internal carotid artery stenosis >70% were included in the study (Supplementary Table 4). Lesion morphology was assessed using a simplified scheme as previously reported [69]. Carotid plaques were characterized as pathologic intimal thickening, fibroatheromas, thin cap fibroatheromas, and ruptures with ulceration or luminal thrombi. Stable plaque phenotypes were generally fibrotic plaques with or without matrix calcification and healed plaque ruptures**.**

Consistent with the method of a previous study [70], each carotid artery segment was collected at the time of endarterectomy en bloc from the intima to the external elastic lamina. Segments collected from the proximal cardiac carotid artery (“normal control”) and at the (plaque-containing) carotid bifurcation (Figure 6A) were snap-frozen within liquid nitrogen and kept at –80°C for qPCR and western blotting. After formalin fixation, ten morphologically complete carotid plaques collected at endarterectomy were embedded in paraffin and serially sectioned throughout the lesion. Histologic sections were prepared at 5 microns, affixed to charged slides, stained for histomorphometric analysis by H&E and modified Movat pentachrome, and underwent immunohistochemistry where CD68 (pan-macrophage marker), MMP9, SPP1, CCL4 and CCL18 were evaluated (Figure 7C).

### Validation of the mRNAs using quantitative real-time polymerase chain reaction (qRT-PCR)

RNAiso Plus reagents (cat. no. 9109, TaKaRa Bio, Inc., Otsu, Japan) were used to extract total RNA from the frozen heart tissues in accordance with the instructions of the manufacturer. One cDNA reverse transcription kit (cat. no. DRR036A, TaKaRa Bio, Inc.) was used to reverse-transcribe RNA, and the SYBR Green PCR kit (cat. no. DRR082A, TaKaRa Bio, Inc.) was used to amplify the resultant cDNA. Then, 2 μg of cDNA was tested in each reaction with the 7500 Fast Real-Time PCR System (Applied Biosystems; Thermo Fisher Scientific). Each experiment was conducted at least three times. The 2^-ΔΔCt^ method was adopted to calculate the expression of genes relative to the housekeeping gene *GAPDH*. Table 2 shows the primers applied to qRT-PCR. The primers used in our study were derived from previous studies [71, 72] or Harvard University's Primer Bank (https://pga.mgh.harvard.edu/primerbank/) and were tested by BLAST analysis before the experiment.

### Identification of potential mRNAs and gene set enrichment analysis (GSEA)

The median value of the expression level of each hub gene was utilized as the cutoff point for dividing data into low and high groups for AS samples at an advanced stage. GSEA [15, 73] was conducted between the two groups to determine the potential functions of these hub genes in advanced-stage AS. The annotated gene set c2.cp.kegg.v6.1.symbols.gmt was selected as the reference gene set. Under the cutoff criterion of NOM *p* value<0.05, the pathways with the top rank of normalized enrichment score (NES) were chosen for analysis.

### Immunohistochemistry

Immunohistochemistry for target proteins was performed by using the Vectastain Elite ABC Kit (Vector Laboratories, CA). Paraffin sections were deparaffinized and rehydrated through a graded alcohol series. Microwave heating at 450 W in 0.01 M EDTA buffer was performed for 20 minutes for antigen retrieval. Incubation with primary antibodies was overnight at 4°C. Control incubations were performed by omitting the specific primary antibody. Slides were counterstained with hematoxylin. The following antibodies were used: pan-macrophage marker CD68 (1:1000; ab213363, Abcam, Cambridge, UK), MMP9 (1:1000; ab76003, Abcam, Cambridge, UK), SPP1 (1:1000; ab214050, Abcam, Cambridge, UK), CCL4 (1:1000, ab235961, Abcam, Cambridge, UK), and CCL18 (1:1000, ab104867, Abcam, Cambridge, UK). Computer-assisted color image analysis segmentation with background correction was used to quantify immunohistochemical staining of macrophages and target proteins.

### Western blotting

Tissue from the ruptured atherosclerotic plaque or control was lysed in ice-cold buffer containing 50 mM Tris-HCl, pH 7.4, 150 mM NaCl, 1% Triton X-100, 1% sodium deoxycholate, 0.1% SDS, sodium orthovanadate, and protease inhibitor cocktail (Beyotime, P1006, Shanghai, CHN) and 1 mM PMSF (Beyotime, ST506, Shanghai, CHN) for 30 minutes. Then, the insoluble material of tissue lysates was removed by centrifugation at 12000x g and 4°C for 30 minutes. After the protein concentration was measured (Beyotime, P0010S, Shanghai, CHN) and normalized to the total protein concentration, the tissue lysate was resuspended in SDS sample buffer and then denatured at 98°C for 5 minutes. Twenty micrograms of total protein was fractionated by electrophoresis on 12% or 10% polyacrylamide gels. The proteins were transferred electrophoretically onto a nitrocellulose membrane (Millipore, IPVH00010, Billerica, MA). After being blocked in 5% nonfat dry milk (BD Difco, 232100, New Jersey, US), the membrane was incubated with the appropriate primary antibodies overnight at 4°C. Proteins were detected by probing western blots with antibodies specific to β-actin (1:5000; ab8227, Abcam, Cambridge, UK), MMP9 (1:1000; ab76003, Abcam, Cambridge, UK), SPP1 (1:1000; ab214050, Abcam, Cambridge, UK), CCL4 (1:1000, ab45690, Abcam, Cambridge, UK), CCL18 (1:1000, ab104867, Abcam, Cambridge, UK), and adipogenesis markers (PPARγ, C/EBP FABP4, 1:1000, CST, Boston, US). Following incubation with horseradish peroxidase-conjugated secondary antibodies (Beyotime, A0208, Shanghai, CHN), the antigen-antibody complexes were detected with an enhanced chemiluminescence detection reagent kit (Thermo, 34577, Massachusetts, US). Protein bands were visualized with a double-color infrared laser imaging system (LI-COR Biotechnology, Nebraska, US). Densitometry analysis of the gels was carried out using ImageJ software from the NIH (http://rsbweb.nih.gov/ij/).

## Supplementary Material

Supplementary Tables

Supplementary Table 1

Supplementary Table 2

Supplementary Table 3

Supplementary Table 5
