# Supplementary material for: Identification and validation of four hub genes involved in the plaque deterioration of atherosclerosis
Source: Aging (Albany NY). 2019 Aug 26;11(16):6469–89. doi: 10.18632/aging.102200 (PMC6738408; doi:10.18632/aging.102200)
Supplement: Supplementary Tables [file aging-11-102200-s005.pdf]

SUPPLEMENTARY TABLES

Please browse Full Text version to see the data of Supplementary Tables 1–3.

Supplementary Table 1. GO result of differentiating plaque sets.

Supplementary Table 2. KEGG PATHWAY result of upregulated genes in differentiating plaque sets.

Supplementary Table 3. KEGG PATHWAY result of poor prognosis sets.

Supplementary Table 4. Sample characteristics in this study.

Parameters	Symptomatic patients	Asymptomatic patients
Age (years)	66.8 (SD 7.2)	65.1 (SD 5.2)
Diabetes (%)	80%	70%
Hypertension (%)	50%	50%
Smoking (past or current, %)	60%	60%
Dyslipidemia (%)	80%	80%
Statin treatment (%)	90%	80%
Fasting lipoproteins (mmol/L):		
Total cholesterol	4.04 (SD 1.06)	3.77 (SD 0.76)
LDL cholesterol	2.39 (SD 0.77)	2.24 (SD 0.32)
HDL cholesterol	0.95 (SD 0.17)	1.01 (SD 0.24)
Triglycerides	1.18 (SD 0.32)	1.33 (SD 0.27)
Creatinin (mmol/L)	77.7 (SD 18.99)	68.3 (SD 12.49)
High sensitive-CRP (mg/L)	4.23 (SD 5.45)	2.84 (SD 1.67)
White blood cell count (109/L)	6.14 (SD 1.55)	5.89 (SD 1.37)

Please browse Full Text version to see the data of Supplementary Table 5.

Supplementary Table 5. The results of differentiating plaque sets and poor prognosis sets using RRA.
