# Supplementary material for: Identification and validation of four hub genes involved in the plaque deterioration of atherosclerosis
Source: Aging (Albany NY). 2019 Aug 26;11(16):6469–89. doi: 10.18632/aging.102200 (PMC6738408; doi:10.18632/aging.102200)
Supplement: Supplementary Table 1 [file aging-11-102200-s004.docx]

| **Supplementary Table 1-1. GO result of differentiating plaque sets** | | | | |  |  |  |  |  |  |  |  |
| --- | --- | --- | --- | --- | --- | --- | --- | --- | --- | --- | --- | --- |
| Category | Term | Count | % | PValue | Genes | List Total | Pop Hits | Pop Total | Fold Enrichment | Bonferroni | Benjamini | FDR |
| CC | GO:0005615~extracellular space | 21 | 0.34 | 7.32E-11 | IBSP, CCL3, MMP9, IL1RN, CHI3L1, MMP7, HP, CCL4, CCL18, FBLN1, CXCL14, SFRP1, APOD, SCRG1, HMOX1, CCL3L3, PLA2G2A, FGF1, MYOC, SPP1, SPON1 | 50 | 1347 | 18224 | 5.68 | 6.66E-09 | 6.66E-09 | 7.98E-08 |
| CC | GO:0005578~proteinaceous extracellular matrix | 10 | 0.16 | 3.46E-08 | FBLN1, SFRP1, MMP9, CHI3L1, MMP7, FGF1, MMP12, MYOC, MMP1, SPON1 | 50 | 268 | 18224 | 13.60 | 3.15E-06 | 1.57E-06 | 3.77E-05 |
| BP | GO:0070374~positive regulation of ERK1 and ERK2 cascade | 8 | 0.13 | 3.85E-07 | CCL3, HAND2, CCL3L3, PLA2G2A, CHI3L1, FGF1, CCL4, CCL18 | 46 | 175 | 16792 | 16.69 | 1.87E-04 | 1.87E-04 | 5.52E-04 |
| BP | GO:0071356~cellular response to tumor necrosis factor | 7 | 0.11 | 4.56E-07 | CYBA, CCL3, SFRP1, CCL3L3, CHI3L1, CCL4, CCL18 | 46 | 110 | 16792 | 23.23 | 2.22E-04 | 1.11E-04 | 6.54E-04 |
| CC | GO:0005576~extracellular region | 18 | 0.29 | 4.68E-07 | IBSP, CCL3, MMP9, MMP7, IL4I1, HP, CCL4, PLAC9, MMP12, MMP1, FBLN1, APOD, SFRP1, CXCL14, CCL3L3, PLA2G2A, FGF1, SPP1 | 50 | 1610 | 18224 | 4.07 | 4.26E-05 | 1.42E-05 | 5.11E-04 |
| BP | GO:0071347~cellular response to interleukin-1 | 6 | 0.10 | 1.25E-06 | CCL3, SFRP1, CCL3L3, CHI3L1, CCL4, CCL18 | 46 | 71 | 16792 | 30.85 | 6.11E-04 | 2.04E-04 | 0.0018 |
| BP | GO:0022617~extracellular matrix disassembly | 6 | 0.10 | 1.76E-06 | MMP9, MMP7, ADAM8, MMP12, MMP1, SPP1 | 46 | 76 | 16792 | 28.82 | 8.58E-04 | 2.15E-04 | 0.002529 |
| MF | GO:0008009~chemokine activity | 5 | 0.08 | 7.11E-06 | CCL3, CXCL14, CCL3L3, CCL4, CCL18 | 44 | 49 | 16881 | 39.15 | 8.39E-04 | 8.39E-04 | 0.00814 |
| BP | GO:0050729~positive regulation of inflammatory response | 5 | 0.08 | 4.28E-05 | CCL3, CCL3L3, PLA2G2A, CCL4, CCL18 | 46 | 73 | 16792 | 25.00 | 0.020626 | 0.00416 | 0.06138 |
| BP | GO:0048247~lymphocyte chemotaxis | 4 | 0.07 | 5.62E-05 | CCL3, CCL3L3, ADAM8, CCL18 | 46 | 28 | 16792 | 52.15 | 0.027008 | 0.004553 | 0.080626 |
| BP | GO:0006954~inflammatory response | 8 | 0.13 | 6.12E-05 | CYBA, CCL3, CCL3L3, CHI3L1, ADAM8, CCL4, CCL18, SPP1 | 46 | 379 | 16792 | 7.71 | 0.029345 | 0.004246 | 0.087706 |
| CC | GO:0031012~extracellular matrix | 7 | 0.11 | 1.36E-04 | IBSP, FBLN1, SFRP1, MMP7, MYOC, MMP1, SPON1 | 50 | 296 | 18224 | 8.62 | 0.012272 | 0.003082 | 0.147824 |
| BP | GO:2000503~positive regulation of natural killer cell chemotaxis | 3 | 0.05 | 1.46E-04 | CCL3, CXCL14, CCL4 | 46 | 7 | 16792 | 156.45 | 0.068736 | 0.008862 | 0.209573 |
| BP | GO:0001649~osteoblast differentiation | 5 | 0.08 | 1.70E-04 | IBSP, CCL3, SFRP1, MYOC, SPP1 | 46 | 104 | 16792 | 17.55 | 0.079544 | 0.009167 | 0.243886 |
| BP | GO:0002548~monocyte chemotaxis | 4 | 0.07 | 1.92E-04 | CCL3, CCL3L3, CCL4, CCL18 | 46 | 42 | 16792 | 34.77 | 0.089231 | 0.009303 | 0.274974 |
| MF | GO:0004222~metalloendopeptidase activity | 5 | 0.08 | 1.92E-04 | MMP9, MMP7, ADAM8, MMP12, MMP1 | 44 | 113 | 16881 | 16.98 | 0.022412 | 0.01127 | 0.219597 |
| MF | GO:0004252~serine-type endopeptidase activity | 6 | 0.10 | 4.56E-04 | MMP9, MMP7, HP, ADAM8, MMP12, MMP1 | 44 | 255 | 16881 | 9.03 | 0.052343 | 0.017761 | 0.520063 |
| BP | GO:0071346~cellular response to interferon-gamma | 4 | 0.07 | 4.76E-04 | CCL3, CCL3L3, CCL4, CCL18 | 46 | 57 | 16792 | 25.62 | 0.206788 | 0.02084 | 0.680164 |
| BP | GO:0030574~collagen catabolic process | 4 | 0.07 | 6.68E-04 | MMP9, MMP7, MMP12, MMP1 | 46 | 64 | 16792 | 22.82 | 0.277918 | 0.02677 | 0.954682 |
| BP | GO:0030593~neutrophil chemotaxis | 4 | 0.07 | 7.31E-04 | CCL3, CCL3L3, CCL4, CCL18 | 46 | 66 | 16792 | 22.12 | 0.299741 | 0.027036 | 1.044187 |
| BP | GO:0070098~chemokine-mediated signaling pathway | 4 | 0.07 | 9.05E-04 | CCL3, CCL3L3, CCL4, CCL18 | 46 | 71 | 16792 | 20.57 | 0.356563 | 0.031004 | 1.290586 |
| BP | GO:0051897~positive regulation of protein kinase B signaling | 4 | 0.07 | 0.001473 | CCL3, CHI3L1, ADAM8, MYOC | 46 | 84 | 16792 | 17.38 | 0.512109 | 0.046718 | 2.092047 |
| BP | GO:0001525~angiogenesis | 5 | 0.08 | 0.002945 | APOD, HAND2, HMOX1, FGF1, ADAM8 | 46 | 223 | 16792 | 8.18 | 0.762219 | 0.085863 | 4.143377 |
| BP | GO:0034605~cellular response to heat | 3 | 0.05 | 0.004406 | HMOX1, FGF1, SCARA5 | 46 | 37 | 16792 | 29.60 | 0.883579 | 0.118828 | 6.139005 |
| BP | GO:0007267~cell-cell signaling | 5 | 0.08 | 0.004683 | CCL3, CXCL14, ADRA2C, CCL4, CCL18 | 46 | 254 | 16792 | 7.19 | 0.898349 | 0.119277 | 6.51339 |
| BP | GO:0006955~immune response | 6 | 0.10 | 0.005182 | HLA-DQB2, CCL3, CXCL14, IL1RN, CCL4, CCL18 | 46 | 421 | 16792 | 5.20 | 0.920349 | 0.124678 | 7.182695 |
| CC | GO:0070062~extracellular exosome | 16 | 0.26 | 0.005531 | CD84, FBLN1, DES, SFRP1, APOD, MMP9, IL1RN, MMP7, CHI3L1, PLA2G2A, ACP5, HP, PI16, IGLL5, MYOC, SPP1 | 50 | 2811 | 18224 | 2.07 | 0.396322 | 0.096015 | 5.86736 |
| BP | GO:0007566~embryo implantation | 3 | 0.05 | 0.005648 | FBLN1, MMP9, SPP1 | 46 | 42 | 16792 | 26.07 | 0.936608 | 0.128833 | 7.804925 |
| MF | GO:0004175~endopeptidase activity | 3 | 0.05 | 0.008339 | MMP9, MMP12, MMP1 | 44 | 54 | 16881 | 21.31 | 0.627746 | 0.218894 | 9.139036 |
| BP | GO:0006508~proteolysis | 6 | 0.10 | 0.010515 | SFRP1, MMP9, MMP7, ADAM8, MMP12, MMP1 | 46 | 500 | 16792 | 4.38 | 0.994188 | 0.217401 | 14.07157 |
| MF | GO:0042802~identical protein binding | 7 | 0.11 | 0.011236 | TRAF1, FBLN1, CCL3, DES, SFRP1, MMP9, CCL4 | 44 | 749 | 16881 | 3.59 | 0.736407 | 0.234074 | 12.13041 |
| BP | GO:0043547~positive regulation of GTPase activity | 6 | 0.10 | 0.017082 | CCL3, SFRP1, CCL3L3, FGF1, CCL4, CCL18 | 46 | 565 | 16792 | 3.88 | 0.999773 | 0.3171 | 21.90139 |
| MF | GO:0031726~CCR1 chemokine receptor binding | 2 | 0.03 | 0.017698 | CCL3, CCL4 | 44 | 7 | 16881 | 109.62 | 0.878408 | 0.296143 | 18.48295 |
| BP | GO:0043305~negative regulation of mast cell degranulation | 2 | 0.03 | 0.018612 | CD84, HMOX1 | 46 | 7 | 16792 | 104.30 | 0.999894 | 0.328207 | 23.62732 |
| BP | GO:0001503~ossification | 3 | 0.05 | 0.019439 | IBSP, MMP9, SPP1 | 46 | 80 | 16792 | 13.69 | 0.99993 | 0.32856 | 24.54524 |
| MF | GO:0031730~CCR5 chemokine receptor binding | 2 | 0.03 | 0.020201 | CCL3, CCL4 | 44 | 8 | 16881 | 95.91 | 0.91002 | 0.291088 | 20.82891 |
| BP | GO:0071456~cellular response to hypoxia | 3 | 0.05 | 0.027306 | SFRP1, HMOX1, ADAM8 | 46 | 96 | 16792 | 11.41 | 0.999999 | 0.416849 | 32.78022 |
| BP | GO:0007229~integrin-mediated signaling pathway | 3 | 0.05 | 0.028902 | IBSP, FBLN1, ADAM8 | 46 | 99 | 16792 | 11.06 | 0.999999 | 0.422666 | 34.34585 |
| BP | GO:0043922~negative regulation by host of viral transcription | 2 | 0.03 | 0.034295 | CCL3, CCL4 | 46 | 13 | 16792 | 56.16 | 1 | 0.467112 | 39.38777 |
| BP | GO:0045730~respiratory burst | 2 | 0.03 | 0.034295 | CYBA, CD52 | 46 | 13 | 16792 | 56.16 | 1 | 0.467112 | 39.38777 |
| BP | GO:0045780~positive regulation of bone resorption | 2 | 0.03 | 0.034295 | ADAM8, SPP1 | 46 | 13 | 16792 | 56.16 | 1 | 0.467112 | 39.38777 |
| CC | GO:0031988~membrane-bounded vesicle | 2 | 0.03 | 0.037005 | IBSP, SPP1 | 50 | 14 | 18224 | 52.07 | 0.967656 | 0.435541 | 33.7064 |
| BP | GO:0045766~positive regulation of angiogenesis | 3 | 0.05 | 0.03802 | HMOX1, CHI3L1, FGF1 | 46 | 115 | 16792 | 9.52 | 1 | 0.490424 | 42.65683 |
| BP | GO:0050900~leukocyte migration | 3 | 0.05 | 0.042312 | CD84, MMP9, MMP1 | 46 | 122 | 16792 | 8.98 | 1 | 0.516172 | 46.2203 |
| BP | GO:0006935~chemotaxis | 3 | 0.05 | 0.042312 | CCL3, CXCL14, CCL18 | 46 | 122 | 16792 | 8.98 | 1 | 0.516172 | 46.2203 |
| BP | GO:0010629~negative regulation of gene expression | 3 | 0.05 | 0.052083 | CCL3, SFRP1, HAND2 | 46 | 137 | 16792 | 7.99 | 1 | 0.580335 | 53.57836 |
| BP | GO:0050850~positive regulation of calcium-mediated signaling | 2 | 0.03 | 0.052283 | CCL3, CCL4 | 46 | 20 | 16792 | 36.50 | 1 | 0.56984 | 53.7185 |
| BP | GO:0051894~positive regulation of focal adhesion assembly | 2 | 0.03 | 0.054826 | SFRP1, MYOC | 46 | 21 | 16792 | 34.77 | 1 | 0.576043 | 55.4685 |
| MF | GO:0048020~CCR chemokine receptor binding | 2 | 0.03 | 0.05701 | CCL3L3, CCL18 | 44 | 23 | 16881 | 33.36 | 0.999019 | 0.579293 | 48.9202 |
| BP | GO:0045671~negative regulation of osteoclast differentiation | 2 | 0.03 | 0.062414 | CCL3, SFRP1 | 46 | 24 | 16792 | 30.42 | 1 | 0.613676 | 60.33194 |
| MF | GO:0008201~heparin binding | 3 | 0.05 | 0.062612 | SFRP1, MMP7, FGF1 | 44 | 160 | 16881 | 7.19 | 0.999514 | 0.571616 | 52.2871 |
| MF | GO:0001968~fibronectin binding | 2 | 0.03 | 0.064208 | FBLN1, MYOC | 44 | 26 | 16881 | 29.51 | 0.999603 | 0.543 | 53.20867 |
| BP | GO:0051928~positive regulation of calcium ion transport | 2 | 0.03 | 0.06744 | CCL3, CCL4 | 46 | 26 | 16792 | 28.08 | 1 | 0.632154 | 63.27585 |
| BP | GO:0032148~activation of protein kinase B activity | 2 | 0.03 | 0.06744 | ADRA2C, FGF1 | 46 | 26 | 16792 | 28.08 | 1 | 0.632154 | 63.27585 |
| BP | GO:0051930~regulation of sensory perception of pain | 2 | 0.03 | 0.06744 | CCL3, ADRA2C | 46 | 26 | 16792 | 28.08 | 1 | 0.632154 | 63.27585 |
| BP | GO:0043392~negative regulation of DNA binding | 2 | 0.03 | 0.072439 | HAND2, HMOX1 | 46 | 28 | 16792 | 26.07 | 1 | 0.64877 | 66.0016 |
| MF | GO:0005125~cytokine activity | 3 | 0.05 | 0.073916 | IL1RN, CCL4, SPP1 | 44 | 176 | 16881 | 6.54 | 0.999884 | 0.561218 | 58.47274 |
| BP | GO:0048662~negative regulation of smooth muscle cell proliferation | 2 | 0.03 | 0.074929 | APOD, HMOX1 | 46 | 29 | 16792 | 25.18 | 1 | 0.651323 | 67.28775 |
| BP | GO:0045765~regulation of angiogenesis | 2 | 0.03 | 0.079889 | SFRP1, HMOX1 | 46 | 31 | 16792 | 23.55 | 1 | 0.665761 | 69.71614 |
| BP | GO:0046676~negative regulation of insulin secretion | 2 | 0.03 | 0.079889 | SFRP1, PDE8B | 46 | 31 | 16792 | 23.55 | 1 | 0.665761 | 69.71614 |
| BP | GO:0031667~response to nutrient levels | 2 | 0.03 | 0.079889 | CYBA, MMP7 | 46 | 31 | 16792 | 23.55 | 1 | 0.665761 | 69.71614 |
| BP | GO:0070555~response to interleukin-1 | 2 | 0.03 | 0.084823 | CYBA, CHI3L1 | 46 | 33 | 16792 | 22.12 | 1 | 0.678889 | 71.96452 |
| CC | GO:0048471~perinuclear region of cytoplasm | 5 | 0.08 | 0.085048 | APOD, HMOX1, PLA2G2A, CHI3L1, SPP1 | 50 | 621 | 18224 | 2.93 | 0.999693 | 0.685095 | 62.05374 |
| BP | GO:0030335~positive regulation of cell migration | 3 | 0.05 | 0.087058 | CCL3, FGF1, MYOC | 46 | 184 | 16792 | 5.95 | 1 | 0.679339 | 72.93086 |
| MF | GO:0005109~frizzled binding | 2 | 0.03 | 0.087816 | SFRP1, MYOC | 44 | 36 | 16881 | 21.31 | 0.999981 | 0.594978 | 65.07228 |
| BP | GO:0007186~G-protein coupled receptor signaling pathway | 6 | 0.10 | 0.091165 | CCL3, SFRP1, CCL3L3, ADRA2C, CCL4, CCL18 | 46 | 899 | 16792 | 2.44 | 1 | 0.687712 | 74.62644 |
| BP | GO:0030198~extracellular matrix organization | 3 | 0.05 | 0.096878 | IBSP, FBLN1, SPP1 | 46 | 196 | 16792 | 5.59 | 1 | 0.701906 | 76.82128 |
| BP | GO:0006953~acute-phase response | 2 | 0.03 | 0.099471 | IL1RN, HP | 46 | 39 | 16792 | 18.72 | 1 | 0.703251 | 77.75798 |
| BP | GO:0000302~response to reactive oxygen species | 2 | 0.03 | 0.099471 | CYBA, APOD | 46 | 39 | 16792 | 18.72 | 1 | 0.703251 | 77.75798 |
| BP | GO:0045668~negative regulation of osteoblast differentiation | 2 | 0.03 | 0.099471 | SFRP1, HAND2 | 46 | 39 | 16792 | 18.72 | 1 | 0.703251 | 77.75798 |

| **Supplementary Table 1-2. GO result of poor prognosis sets** | | | | | | | | | |  |  |  |
| --- | --- | --- | --- | --- | --- | --- | --- | --- | --- | --- | --- | --- |
| Category | Term | Count | % | PValue | Genes | List Total | Pop Hits | Pop Total | Fold Enrichment | Bonferroni | Benjamini | FDR |
| BP | GO:0006955~immune response | 24 | 0.14 | 2.40E-14 | GPR183, C5AR1, CCL2, AQP9, IGLV1-44, NCF4, JCHAIN, CTSS, ACKR4, HLA-DMB, HLA-DMA, C1QC, CCL4, HLA-DQA1, CD74, CCL18, FCGR2B, TNFSF13B, FCGR1B, IGHV3-23, IGHA2, IGKC, FCGR3B, IGLC1 | 121 | 421 | 16792 | 7.91 | 2.11E-11 | 2.11E-11 | 3.74E-11 |
| CC | GO:0005576~extracellular region | 41 | 0.24 | 8.77E-13 | CCL2, IGLV1-44, COL21A1, MMP9, NPNT, F13A1, S100A9, JCHAIN, CSPG4, IFI30, MMRN1, CCL4, C1QC, NOV, OGN, CRISPLD1, APOE, CCL3L3, IGHV3-23, PYCARD, IGHA2, IGKC, TPSB2, FIBIN, PLTP, SPP1, CTSS, TCN2, MMP12, C1QA, C1QB, COL14A1, TNFSF13B, CXCL16, PLA2G7, TPSAB1, TREM2, MFAP4, ADAM12, IGLC1, CD14 | 131 | 1610 | 18224 | 3.54 | 1.52E-10 | 1.52E-10 | 1.07E-9 |
| BP | GO:0006954~inflammatory response | 21 | 0.12 | 2.68E-12 | C3AR1, CCL2, C5AR1, TSPAN2, LY96, HCK, LY86, S100A9, ACKR1, FPR3, ITGB2, CCL4, CD180, CCL18, CCRL2, CXCR4, CCL3L3, PYCARD, CD14, SPP1, AOC3 | 121 | 379 | 16792 | 7.69 | 2.35E-9 | 1.17E-9 | 4.16E-9 |
| BP | GO:0045087~innate immune response | 18 | 0.11 | 1.08E-8 | LY96, LY86, S100A9, JCHAIN, C1QC, CD180, C1QA, C1QB, CORO1A, IGHV3-23, PYCARD, FCER1G, IGHA2, IGKC, TREM2, IGLC1, CD14, TYROBP | 121 | 430 | 16792 | 5.81 | 9.49E-6 | 3.16E-6 | 1.68E-5 |
| BP | GO:0030593~neutrophil chemotaxis | 9 | 0.05 | 2.18E-8 | CCL2, C5AR1, CCL3L3, S100A9, ITGA1, FCER1G, ITGB2, CCL4, CCL18 | 121 | 66 | 16792 | 18.92 | 1.91E-5 | 4.79E-6 | 3.39E-5 |
| BP | GO:0070374~positive regulation of ERK1 and ERK2 cascade | 12 | 0.07 | 4.99E-8 | GPR183, CCL2, C5AR1, ARRB2, NPNT, CCL3L3, PYCARD, TREM2, HTR2B, CCL4, CD74, CCL18 | 121 | 175 | 16792 | 9.52 | 4.38E-5 | 8.76E-6 | 7.75E-5 |
| CC | GO:0005615~extracellular space | 30 | 0.18 | 6.08E-8 | CCL2, LY86, MMP9, S100A9, JCHAIN, C1QC, CCL4, OGN, APOE, HMOX1, CCL3L3, C1QTNF2, IGHV3-23, IGHA2, IGKC, PLTP, SPP1, LY96, ACTA2, CTSS, TCN2, CCL18, COL14A1, TNFSF13B, CPE, CXCL16, TPSAB1, IGLC1, CD14, LCP1 | 131 | 1347 | 18224 | 3.10 | 1.05E-5 | 5.29E-6 | 7.44E-5 |
| CC | GO:0005886~plasma membrane | 57 | 0.33 | 1.58E-7 | ADORA3, AQP9, IGLV1-44, LY86, S100A9, ANO1, CCRL2, CXCR4, APOE, HMOX1, SLC22A3, PIK3AP1, FCGR3B, LAIR1, C5AR1, LY96, ACKR1, TNFRSF17, ACKR4, HLA-DQA1, TNFSF13B, ARRB2, CPE, VAMP8, CNTN3, ADAM12, TREM2, LCP1, AOC3, GPR183, C3AR1, ITGA10, IFI30, FPR3, ITGB2, MRAP2, CD72, CD74, RAC2, IL10RA, PCDHB16, IGHV3-23, FCGR1B, FCER1G, IGKC, TYROBP, ITGA1, TSPAN13, SLCO2B1, CORO1A, TMEM47, ADAP2, FCGR2B, CXCL16, HTR2B, IGLC1, CD14 | 131 | 4121 | 18224 | 1.92 | 2.75E-5 | 9.17E-6 | 1.93E-4 |
| BP | GO:0070098~chemokine-mediated signaling pathway | 8 | 0.05 | 7.27E-7 | CCRL2, CCL2, CXCR4, CCL3L3, ACKR1, ACKR4, CCL4, CCL18 | 121 | 71 | 16792 | 15.64 | 6.3806753 | 1.0637287 | 0.0011295 |
| BP | GO:0006898~receptor-mediated endocytosis | 11 | 0.06 | 8.83E-7 | IGLV1-44, APOE, CXCL16, FCGR1B, IGHV3-23, JCHAIN, IGHA2, ACKR4, IGKC, IGLC1, CD14 | 121 | 186 | 16792 | 8.21 | 7.7538054 | 1.1080547 | 0.0013726 |
| MF | GO:0004252~serine-type endopeptidase activity | 12 | 0.07 | 1.03E-6 | C1QA, C1QB, IGLV1-44, MMP9, IGHV3-23, CTSS, IGKC, TPSB2, TPSAB1, IGLC1, C1QC, MMP12 | 113 | 255 | 16881 | 7.03 | 2.65E-4 | 2.65E-4 | 0.0013448 |
| CC | GO:0070062~extracellular exosome | 43 | 0.25 | 1.13E-6 | RARRES1, MMP9, NPNT, S100A9, ANO1, JCHAIN, CSPG4, ACP5, ITGB2, HLA-DMA, C1QC, CD74, OGN, EFHD1, PFN2, RAC2, CRISPLD1, CXCR4, APOE, IGHV3-23, IGHA2, IGKC, FCGR3B, SPP1, LAIR1, ACTA2, ITGA1, GSTT2, TCN2, C1QA, C1QB, CORO1A, COL14A1, CPE, VAMP8, CAPG, FCGBP, MFAP4, VMO1, VSIG4, IGLC1, LCP1, CD14 | 131 | 2811 | 18224 | 2.13 | 1.95E-4 | 4.89E-5 | 0.0013792 |
| BP | GO:0006935~chemotaxis | 9 | 0.05 | 2.63E-6 | CCRL2, C3AR1, CCL2, C5AR1, RAC2, CXCR4, CXCL16, ACKR4, CCL18 | 121 | 122 | 16792 | 10.24 | 0.0023028 | 2.88E-4 | 0.00407994 |
| BP | GO:0006911~phagocytosis, engulfment | 6 | 0.04 | 4.69E-6 | IGHV3-23, FCER1G, IGHA2, IGKC, TREM2, IGLC1 | 121 | 35 | 16792 | 23.79 | 0.0041070 | 4.57E-4 | 0.00728272 |
| BP | GO:0006958~complement activation, classical pathway | 8 | 0.05 | 6.90E-6 | C1QA, C1QB, IGLV1-44, IGHV3-23, IGHA2, IGKC, IGLC1, C1QC | 121 | 99 | 16792 | 11.21 | 0.0060408 | 6.05E-4 | 0.01072202 |
| CC | GO:0072562~blood microparticle | 9 | 0.05 | 1.34E-5 | C1QB, APOE, F13A1, IGHV3-23, JCHAIN, IGHA2, IGKC, IGLC1, C1QC | 131 | 152 | 18224 | 8.24 | 0.0023279 | 4.66024034 | 0.01640400 |
| BP | GO:0022617~extracellular matrix disassembly | 7 | 0.04 | 1.71E-5 | MMP9, CAPG, CTSS, TPSAB1, MMP12, LCP1, SPP1 | 121 | 76 | 16792 | 12.78 | 0.0148808 | 0.00136204 | 0.02652838 |
| MF | GO:0034987~immunoglobulin receptor binding | 5 | 0.03 | 2.45E-5 | IGHV3-23, JCHAIN, IGHA2, IGKC, IGLC1 | 113 | 26 | 16881 | 28.73 | 0.0063075 | 0.00315877 | 0.03201354 |
| BP | GO:0006956~complement activation | 7 | 0.04 | 3.70E-5 | C1QA, C1QB, IGLV1-44, IGHV3-23, IGKC, IGLC1, C1QC | 121 | 87 | 16792 | 11.17 | 0.0319671 | 0.00270377 | 0.05747838 |
| BP | GO:0050776~regulation of immune response | 9 | 0.05 | 4.17E-5 | LAIR1, FCGR2B, IGLV1-44, IGHV3-23, ITGB2, IGKC, TREM2, IGLC1, TYROBP | 121 | 178 | 16792 | 7.02 | 0.0359502 | 0.00281237 | 0.06477041 |
| MF | GO:0019864~IgG binding | 4 | 0.02 | 4.51E-5 | FCGR2B, FCGR1B, FCER1G, FCGR3B | 113 | 11 | 16881 | 54.32 | 0.0115758 | 0.00387358 | 0.05890020 |
| MF | GO:0005518~collagen binding | 6 | 0.04 | 4.80E-5 | ASPN, COL14A1, MMP9, ITGA1, ITGA10, CTSS | 113 | 60 | 16881 | 14.94 | 0.0123062 | 0.0030908 | 0.0626386 |
| BP | GO:0019886~antigen processing and presentation of exogenous peptide antigen via MHC class II | 7 | 0.04 | 5.08E-5 | IFI30, FCER1G, CTSS, HLA-DMB, HLA-DMA, CD74, HLA-DQA1 | 121 | 92 | 16792 | 10.56 | 0.0435874 | 0.0031782 | 0.0788352 |
| BP | GO:0007229~integrin-mediated signaling pathway | 7 | 0.04 | 7.66E-5 | HCK, ITGA1, FCER1G, ITGA10, ITGB2, ADAM12, TYROBP | 121 | 99 | 16792 | 9.81 | 0.0650359 | 0.0044731 | 0.1189336 |
| BP | GO:0060326~cell chemotaxis | 6 | 0.04 | 1.00E-4 | NOV, C3AR1, CCL2, C5AR1, ARRB2, FPR3 | 121 | 65 | 16792 | 12.81 | 0.08448872 | 0.00550184 | 0.1560903 |
| BP | GO:0007155~cell adhesion | 13 | 0.08 | 1.10E-4 | CCL2, HCK, ITGA10, ITGB2, MMRN1, CD72, CCL4, SUSD5, CNTN3, MFAP4, ADAM12, SPP1, AOC3 | 121 | 459 | 16792 | 3.93 | 0.09212929 | 0.00566935 | 0.1708970 |
| BP | GO:0050729~positive regulation of inflammatory response | 6 | 0.04 | 1.75E-4 | CCL2, CCL3L3, S100A9, PLA2G7, CCL4, CCL18 | 121 | 73 | 16792 | 11.41 | 0.14220840 | 0.00848568 | 0.2710869 |
| BP | GO:0050900~leukocyte migration | 7 | 0.04 | 2.42E-4 | C3AR1, C5AR1, MMP9, FCER1G, FPR3, ITGB2, CD74 | 121 | 122 | 16792 | 7.96 | 0.19132034 | 0.01111421 | 0.3750859 |
| BP | GO:0006508~proteolysis | 13 | 0.08 | 2.43E-4 | C1QA, C1QB, IGLV1-44, MMP9, IGHV3-23, CTSS, ADAM12, IGKC, TPSB2, TPSAB1, IGLC1, C1QC, MMP12 | 121 | 500 | 16792 | 3.61 | 0.19236673 | 0.01062550 | 0.3773686 |
| BP | GO:0034142~toll-like receptor 4 signaling pathway | 4 | 0.02 | 2.69E-4 | LY96, PIK3AP1, ITGB2, CD14 | 121 | 18 | 16792 | 30.84 | 0.21006777 | 0.01116614 | 0.4164302 |
| CC | GO:0031012~extracellular matrix | 10 | 0.06 | 2.76E-4 | ASPN, NOV, OGN, COL14A1, COL21A1, APOE, S100A9, MMRN1, MFAP4, TPSAB1 | 131 | 296 | 18224 | 4.70 | 0.04686170 | 0.00796730 | 0.3372648 |
| MF | GO:0008009~chemokine activity | 5 | 0.03 | 3.09E-4 | CCL2, CXCL16, CCL3L3, CCL4, CCL18 | 113 | 49 | 16881 | 15.24 | 0.07665752 | 0.01582447 | 0.4027633 |
| BP | GO:0032760~positive regulation of tumor necrosis factor production | 5 | 0.03 | 3.49E-4 | CCL2, LY96, PYCARD, FCER1G, CD14 | 121 | 47 | 16792 | 14.76 | 0.26392037 | 0.01383149 | 0.5407856 |
| CC | GO:0043202~lysosomal lumen | 6 | 0.04 | 3.55E-4 | OGN, CSPG4, IFI30, CTSS, TCN2, CD74 | 131 | 85 | 18224 | 9.82 | 0.05995941 | 0.00879427 | 0.4342863 |
| CC | GO:0042613~MHC class II protein complex | 4 | 0.02 | 4.95E-4 | HLA-DMB, HLA-DMA, CD74, HLA-DQA1 | 131 | 22 | 18224 | 25.29 | 0.08248481 | 0.01070307 | 0.6041213 |
| BP | GO:0090023~positive regulation of neutrophil chemotaxis | 4 | 0.02 | 4.96E-4 | C3AR1, C5AR1, RAC2, CD74 | 121 | 22 | 16792 | 25.23 | 0.35331416 | 0.01877348 | 0.7684169 |
| CC | GO:0005581~collagen trimer | 6 | 0.04 | 5.12E-4 | C1QA, C1QB, COL14A1, COL21A1, C1QTNF2, C1QC | 131 | 92 | 18224 | 9.07 | 0.08524460 | 0.00985100 | 0.6251952 |
| MF | GO:0003823~antigen binding | 6 | 0.04 | 6.14E-4 | IGLV1-44, IGHV3-23, JCHAIN, IGHA2, IGKC, IGLC1 | 113 | 103 | 16881 | 8.70 | 0.14648142 | 0.02605261 | 0.7982716 |
| BP | GO:0050871~positive regulation of B cell activation | 4 | 0.02 | 8.21E-4 | IGHV3-23, IGHA2, IGKC, IGLC1 | 121 | 26 | 16792 | 21.35 | 0.51367251 | 0.02958976 | 1.2675961 |
| BP | GO:0000187~activation of MAPK activity | 6 | 0.04 | 0.0010197 | C5AR1, CXCR4, C1QTNF2, CSPG4, ITGA1, CD74 | 121 | 107 | 16792 | 7.78 | 0.59173042 | 0.03519869 | 1.5728085 |
| BP | GO:0006910~phagocytosis, recognition | 4 | 0.02 | 0.0010233 | IGHV3-23, IGHA2, IGKC, IGLC1 | 121 | 28 | 16792 | 19.83 | 0.59301938 | 0.03398559 | 1.5783162 |
| CC | GO:0005887~integral component of plasma membrane | 22 | 0.13 | 0.0010333 | GPR183, C3AR1, C5AR1, AQP9, TSPAN2, CSPG4, TSPAN13, CD52, FPR3, ACKR4, SLCO2B1, CD72, HLA-DQA1, CD180, CCRL2, LAPTM5, GPR34, SLC16A6, SLC22A3, FCER1G, HTR2B, TYROBP | 131 | 1415 | 18224 | 2.16 | 0.16464011 | 0.01782842 | 1.2582667 |
| BP | GO:0071356~cellular response to tumor necrosis factor | 6 | 0.04 | 0.0011543 | CCL2, NPNT, CCL3L3, PYCARD, CCL4, CCL18 | 121 | 110 | 16792 | 7.57 | 0.63726547 | 0.03686208 | 1.7785751 |
| BP | GO:0007200~phospholipase C-activating G-protein coupled receptor signaling pathway | 5 | 0.03 | 0.0012703 | C3AR1, C5AR1, ANO1, FPR3, HTR2B | 121 | 66 | 16792 | 10.51 | 0.67243030 | 0.03907516 | 1.9556585 |
| BP | GO:0031666~positive regulation of lipopolysaccharide-mediated signaling pathway | 3 | 0.02 | 0.0013787 | LY96, LY86, CD180 | 121 | 8 | 16792 | 52.04 | 0.702214009 | 0.04091131 | 2.1209140 |
| CC | GO:0016021~integral component of membrane | 54 | 0.32 | 0.0014933 | ADORA3, RARRES1, TSPAN2, AQP9, MPEG1, ANO1, CSPG4, CD52, HLA-DMB, HLA-DMA, CCRL2, CXCR4, HMOX1, SLC22A3, FCGR3B, LAIR1, MS4A7, TMEM255A, ACKR1, TNFRSF17, HLA-DQA1, TNFSF13B, VAMP8, SUSD5, ADAM12, VSIG4, TREM2, AOC3, GPR183, C3AR1, ACP5, FPR3, MRAP2, CD72, CD74, PFN2, IL10RA, PCDHB16, FCGR1B, FCER1G, TYROBP, MS4A4A, ST6GAL2, TSPAN13, SLCO2B1, TMEM47, GPR34, FCGR2B, UCP2, SLC16A6, CXCL16, PLN, HTR2B, TMEM176A | 131 | 5163 | 18224 | 1.46 | 0.22897539 | 0.02336232 | 1.8137161 |
| BP | GO:0031663~lipopolysaccharide-mediated signaling pathway | 4 | 0.02 | 0.0015175 | CCL2, LY96, HCK, CD14 | 121 | 32 | 16792 | 17.35 | 0.73642917 | 0.04347444 | 2.3320968 |
| BP | GO:0071347~cellular response to interleukin-1 | 5 | 0.03 | 0.0016663 | CCL2, CCL3L3, PYCARD, CCL4, CCL18 | 121 | 71 | 16792 | 9.77 | 0.76875248 | 0.04613622 | 2.5579663 |
| BP | GO:0010759~positive regulation of macrophage chemotaxis | 3 | 0.02 | 0.0026705 | C3AR1, CCL2, C5AR1 | 121 | 11 | 16792 | 37.85 | 0.90442578 | 0.07074341 | 4.0697626 |
| BP | GO:0002523~leukocyte migration involved in inflammatory response | 3 | 0.02 | 0.0026705 | CCL2, S100A9, ITGB2 | 121 | 11 | 16792 | 37.85 | 0.90442578 | 0.07074341 | 4.0697626 |
| CC | GO:0005578~proteinaceous extracellular matrix | 8 | 0.05 | 0.0031314 | ASPN, NOV, OGN, COL14A1, COL21A1, MMP9, NPNT, MMP12 | 131 | 268 | 18224 | 4.15 | 0.42057581 | 0.04445813 | 3.7684411 |
| BP | GO:0002548~monocyte chemotaxis | 4 | 0.02 | 0.0033349 | CCL2, CCL3L3, CCL4, CCL18 | 121 | 42 | 16792 | 13.22 | 0.94676068 | 0.08504237 | 5.0579355 |
| BP | GO:0002250~adaptive immune response | 6 | 0.04 | 0.004207 | GPR183, LAIR1, FCGR1B, JCHAIN, TNFRSF17, CTSS | 121 | 148 | 16792 | 5.63 | 0.97533509 | 0.10317394 | 6.3419081 |
| CC | GO:0009897~external side of plasma membrane | 7 | 0.04 | 0.004266 | IGHV3-23, ITGA1, FCER1G, IGHA2, IGKC, IGLC1, CD74 | 131 | 213 | 18224 | 4.57 | 0.52476933 | 0.05562061 | 5.1018902 |
| MF | GO:0023026~MHC class II protein complex binding | 3 | 0.02 | 0.004926 | HLA-DMB, HLA-DMA, CD74 | 113 | 16 | 16881 | 28.01 | 0.72036103 | 0.16642913 | 6.2445043 |
| CC | GO:0005769~early endosome | 7 | 0.04 | 0.006041 | CORO1A, VAMP8, APOE, CXCR4, ACKR1, ACKR4, AOC3 | 131 | 229 | 18224 | 4.25 | 0.65159116 | 0.07254663 | 7.1529690 |
| BP | GO:0002504~antigen processing and presentation of peptide or polysaccharide antigen via MHC class II | 3 | 0.02 | 0.006421 | HLA-DMB, HLA-DMA, HLA-DQA1 | 121 | 17 | 16792 | 24.49 | 0.99650328 | 0.14921689 | 9.5244599 |
| BP | GO:0050853~B cell receptor signaling pathway | 4 | 0.02 | 0.006772 | IGHV3-23, IGHA2, IGKC, IGLC1 | 121 | 54 | 16792 | 10.28 | 0.99743635 | 0.15272610 | 10.020071 |
| BP | GO:0019882~antigen processing and presentation | 4 | 0.02 | 0.007126 | CTSS, HLA-DMB, CD74, HLA-DQA1 | 121 | 55 | 16792 | 10.09 | 0.99812510 | 0.15608891 | 10.516897 |
| BP | GO:0006959~humoral immune response | 4 | 0.02 | 0.007866 | GPR183, CCL2, LY86, TREM2 | 121 | 57 | 16792 | 9.74 | 0.99902589 | 0.16679321 | 11.547801 |
| BP | GO:0071346~cellular response to interferon-gamma | 4 | 0.02 | 0.007866 | CCL2, CCL3L3, CCL4, CCL18 | 121 | 57 | 16792 | 9.74 | 0.99902589 | 0.16679321 | 11.547801 |
| CC | GO:0042571~immunoglobulin complex, circulating | 3 | 0.02 | 0.007975 | IGHV3-23, IGKC, IGLC1 | 131 | 19 | 18224 | 21.97 | 0.75174934 | 0.08870421 | 9.3418573 |
| BP | GO:0071222~cellular response to lipopolysaccharide | 5 | 0.03 | 0.008784 | CCL2, CXCL16, PYCARD, CD14, CD180 | 121 | 113 | 16792 | 6.14 | 0.99956777 | 0.18014690 | 12.810638 |
| MF | GO:0005102~receptor binding | 8 | 0.05 | 0.009173 | CCL2, TNFSF13B, ARRB2, HCK, C1QTNF2, ITGA1, CD72, TYROBP | 113 | 353 | 16881 | 3.39 | 0.90722509 | 0.25710263 | 11.335470 |
| MF | GO:0048020~CCR chemokine receptor binding | 3 | 0.02 | 0.010078 | CCRL2, CCL3L3, CCL18 | 113 | 23 | 16881 | 19.49 | 0.92671195 | 0.25201554 | 12.387030 |
| CC | GO:0005884~actin filament | 4 | 0.02 | 0.011230 | CORO1A, RAC2, HCK, LCP1 | 131 | 65 | 18224 | 8.56 | 0.85987047 | 0.11558091 | 12.918700 |
| BP | GO:0007186~G-protein coupled receptor signaling pathway | 14 | 0.08 | 0.011980 | CCRL2, C3AR1, GPR183, CCL2, GPR34, RAC2, CXCR4, APOE, CCL3L3, ACKR1, ACKR4, HTR2B, CCL4, CCL18 | 121 | 899 | 16792 | 2.16 | 0.99997463 | 0.23244809 | 17.077571 |
| BP | GO:0038096~Fc-gamma receptor signaling pathway involved in phagocytosis | 5 | 0.03 | 0.013070 | IGLV1-44, HCK, IGHV3-23, IGKC, IGLC1 | 121 | 127 | 16792 | 5.46 | 0.99999037 | 0.24552756 | 18.487735 |
| BP | GO:0030198~extracellular matrix organization | 6 | 0.04 | 0.013324 | COL14A1, NPNT, ITGA1, ITGA10, ITGB2, SPP1 | 121 | 196 | 16792 | 4.25 | 0.99999232 | 0.24454000 | 18.814231 |
| CC | GO:0005602~complement component C1 complex | 2 | 0.01 | 0.014216 | C1QA, C1QB | 131 | 2 | 18224 | 139.11 | 0.91720651 | 0.13632029 | 16.085190 |
| BP | GO:0042590~antigen processing and presentation of exogenous peptide antigen via MHC class I | 2 | 0.01 | 0.014241 | IFI30, FCER1G | 121 | 2 | 16792 | 138.78 | 0.99999660 | 0.25389566 | 19.978542 |
| BP | GO:0002588~positive regulation of antigen processing and presentation of peptide antigen via MHC class II | 2 | 0.01 | 0.014241 | PYCARD, TREM2 | 121 | 2 | 16792 | 138.78 | 0.99999660 | 0.25389566 | 19.978542 |
| BP | GO:0060333~interferon-gamma-mediated signaling pathway | 4 | 0.02 | 0.014299 | HCK, FCGR1B, IFI30, HLA-DQA1 | 121 | 71 | 16792 | 7.82 | 0.99999677 | 0.24978094 | 20.050588 |
| MF | GO:0004872~receptor activity | 6 | 0.04 | 0.014832 | IL10RA, ACKR1, TNFRSF17, ITGB2, TREM2, CD180 | 113 | 217 | 16881 | 4.13 | 0.97883742 | 0.31992492 | 17.724522 |
| BP | GO:0043524~negative regulation of neuron apoptotic process | 5 | 0.03 | 0.014873 | CORO1A, CCL2, C5AR1, APOE, HMOX1 | 121 | 132 | 16792 | 5.26 | 0.99999806 | 0.25351791 | 20.771854 |
| BP | GO:0007204~positive regulation of cytosolic calcium ion concentration | 5 | 0.03 | 0.015637 | C3AR1, C5AR1, CXCR4, CD52, FPR3 | 121 | 134 | 16792 | 5.18 | 0.99999902 | 0.25979113 | 21.720361 |
| CC | GO:0009986~cell surface | 10 | 0.06 | 0.015701 | C5AR1, CXCR4, CSPG4, ITGA1, FCER1G, ITGB2, HLA-DMA, CD74, TYROBP, AOC3 | 131 | 542 | 18224 | 2.57 | 0.93631113 | 0.14185814 | 17.620532 |
| CC | GO:0008305~integrin complex | 3 | 0.02 | 0.015774 | ITGA1, ITGA10, ITGB2 | 131 | 27 | 18224 | 15.46 | 0.93712526 | 0.13550616 | 17.695099 |
| BP | GO:0048247~lymphocyte chemotaxis | 3 | 0.02 | 0.016958 | CCL2, CCL3L3, CCL18 | 121 | 28 | 16792 | 14.87 | 0.99999969 | 0.27349364 | 23.336723 |
| BP | GO:0007165~signal transduction | 16 | 0.09 | 0.018404 | C5AR1, CCL2, S100A9, TNFRSF17, FPR3, CD72, CCL4, CCL18, CD74, FCGR2B, RAC2, TNFSF13B, ARRB2, PYCARD, CHN1, TYROBP | 121 | 1161 | 16792 | 1.91 | 0.99999991 | 0.28808315 | 25.071310 |
| BP | GO:0042742~defense response to bacterium | 5 | 0.03 | 0.020275 | IGHV3-23, S100A9, FCER1G, IGKC, IGLC1 | 121 | 145 | 16792 | 4.79 | 0.99999998 | 0.30721178 | 27.258956 |
| BP | GO:0050731~positive regulation of peptidyl-tyrosine phosphorylation | 4 | 0.02 | 0.020943 | ARRB2, CSPG4, TREM2, CD74 | 121 | 82 | 16792 | 6.77 | 0.99999999 | 0.31041659 | 28.025786 |
| CC | GO:0071748~monomeric IgA immunoglobulin complex | 2 | 0.01 | 0.021249 | JCHAIN, IGHA2 | 131 | 3 | 18224 | 92.74 | 0.97617957 | 0.17044061 | 23.130372 |
| CC | GO:0071751~secretory IgA immunoglobulin complex | 2 | 0.01 | 0.021249 | JCHAIN, IGHA2 | 131 | 3 | 18224 | 92.74 | 0.97617957 | 0.17044061 | 23.130372 |
| CC | GO:0071752~secretory dimeric IgA immunoglobulin complex | 2 | 0.01 | 0.021249 | JCHAIN, IGHA2 | 131 | 3 | 18224 | 92.74 | 0.97617957 | 0.17044061 | 23.130372 |
| CC | GO:0005764~lysosome | 6 | 0.04 | 0.023057 | LAPTM5, CXCR4, HCK, IFI30, ACP5, CTSS | 131 | 226 | 18224 | 3.69 | 0.98273195 | 0.17574952 | 24.851388 |
| MF | GO:0070891~lipoteichoic acid binding | 2 | 0.01 | 0.026278 | TREM2, CD14 | 113 | 4 | 16881 | 74.69 | 0.99896195 | 0.46451542 | 29.365872 |
| BP | GO:0007160~cell-matrix adhesion | 4 | 0.02 | 0.026671 | NPNT, ITGA1, ITGA10, ITGB2 | 121 | 90 | 16792 | 6.17 | 0.99999999 | 0.37211800 | 34.297823 |
| MF | GO:0042802~identical protein binding | 11 | 0.06 | 0.027114 | APOE, MMP9, PLN, C1QTNF2, PYCARD, PIK3AP1, MRAP2, CCL4, LCP1, CD74, TYROBP | 113 | 749 | 16881 | 2.19 | 0.99916839 | 0.44623409 | 30.154006 |
| CC | GO:0030485~smooth muscle contractile fiber | 2 | 0.01 | 0.028232 | ACTA2, NPNT | 131 | 4 | 18224 | 69.56 | 0.99314711 | 0.20268378 | 29.584407 |
| BP | GO:0032497~detection of lipopolysaccharide | 2 | 0.01 | 0.028282 | LY96, TREM2 | 121 | 4 | 16792 | 69.39 | 0.99999999 | 0.38394785 | 35.967369 |
| BP | GO:0045123~cellular extravasation | 2 | 0.01 | 0.028282 | ITGA1, ITGB2 | 121 | 4 | 16792 | 69.39 | 0.99999999 | 0.38394785 | 35.967369 |
| BP | GO:0002283~neutrophil activation involved in immune response | 2 | 0.01 | 0.028282 | FCER1G, TYROBP | 121 | 4 | 16792 | 69.39 | 0.99999999 | 0.38394785 | 35.967369 |
| BP | GO:0006874~cellular calcium ion homeostasis | 4 | 0.02 | 0.029014 | CCL2, APOE, PLN, HTR2B | 121 | 93 | 16792 | 5.97 | 0.99999999 | 0.38600172 | 36.712490 |
| CC | GO:0030658~transport vesicle membrane | 3 | 0.02 | 0.030029 | CPE, CD74, HLA-DQA1 | 131 | 38 | 18224 | 10.98 | 0.99503438 | 0.20599192 | 31.163087 |
| BP | GO:0030890~positive regulation of B cell proliferation | 3 | 0.02 | 0.031597 | GPR183, TNFSF13B, CD74 | 121 | 39 | 16792 | 10.68 | 0.99999999 | 0.406693796 | 39.278590 |
| MF | GO:0098639~collagen binding involved in cell-matrix adhesion | 2 | 0.01 | 0.032739 | ITGA1, ITGA10 | 113 | 5 | 16881 | 59.76 | 0.99981372 | 0.48347751 | 35.246593 |
| BP | GO:0001895~retina homeostasis | 3 | 0.02 | 0.033107 | JCHAIN, IGHA2, IGKC | 121 | 40 | 16792 | 10.41 | 0.99999999 | 0.41577582 | 40.733527 |
| CC | GO:0030669~clathrin-coated endocytic vesicle membrane | 3 | 0.02 | 0.034547 | FCGR1B, CD74, HLA-DQA1 | 131 | 41 | 18224 | 10.18 | 0.99779626 | 0.22500600 | 34.988953 |
| BP | GO:0002503~peptide antigen assembly with MHC class II protein complex | 2 | 0.01 | 0.035228 | HLA-DMB, HLA-DMA | 121 | 5 | 16792 | 55.51 | 0.99999999 | 0.43009994 | 42.721129 |
| BP | GO:0007267~cell-cell signaling | 6 | 0.04 | 0.035721 | NOV, C1QA, S100A9, ITGB2, CCL4, CCL18 | 121 | 254 | 16792 | 3.28 | 0.99999999 | 0.42896119 | 43.173960 |
| BP | GO:0031623~receptor internalization | 3 | 0.02 | 0.037804 | ARRB2, FCER1G, ITGB2 | 121 | 43 | 16792 | 9.68 | 0.99999999 | 0.44198801 | 45.051668 |
| BP | GO:0038095~Fc-epsilon receptor signaling pathway | 5 | 0.03 | 0.038879 | IGLV1-44, IGHV3-23, FCER1G, IGKC, IGLC1 | 121 | 178 | 16792 | 3.90 | 0.99999999 | 0.44574738 | 45.998511 |
| BP | GO:0030838~positive regulation of actin filament polymerization | 3 | 0.02 | 0.041067 | PFN2, HCK, PYCARD | 121 | 45 | 16792 | 9.25 | 0.99999999 | 0.45862826 | 47.877288 |
| CC | GO:0046696~lipopolysaccharide receptor complex | 2 | 0.01 | 0.042050 | LY96, CD14 | 131 | 6 | 18224 | 46.37 | 0.99943293 | 0.25844180 | 40.913172 |
| BP | GO:0060267~positive regulation of respiratory burst | 2 | 0.01 | 0.042124 | JCHAIN, IGHA2 | 121 | 6 | 16792 | 46.26 | 1.0 | 0.46176715 | 48.762886 |
| BP | GO:0031100~organ regeneration | 3 | 0.02 | 0.044433 | CCL2, C5AR1, LCP1 | 121 | 47 | 16792 | 8.86 | 1.0 | 0.47462743 | 50.648518 |
| BP | GO:0006909~phagocytosis | 3 | 0.02 | 0.046153 | CORO1A, ITGB2, CD14 | 121 | 48 | 16792 | 8.67 | 1.0 | 0.48239393 | 52.010932 |
| CC | GO:0005765~lysosomal membrane | 6 | 0.04 | 0.046688 | LAPTM5, VAMP8, HLA-DMB, HLA-DMA, CD74, HLA-DQA1 | 131 | 274 | 18224 | 3.05 | 0.99975631 | 0.27384069 | 44.323514 |
| BP | GO:0045766~positive regulation of angiogenesis | 4 | 0.02 | 0.049381 | C3AR1, C5AR1, HMOX1, ITGB2 | 121 | 115 | 16792 | 4.83 | 1.0 | 0.50080087 | 54.472832 |
| CC | GO:1903561~extracellular vesicle | 3 | 0.02 | 0.049526 | OGN, APOE, ITGB2 | 131 | 50 | 18224 | 8.35 | 0.99985493 | 0.27916563914877324 | 46.319676 |
| BP | GO:0010818~T cell chemotaxis | 2 | 0.01 | 0.055771 | GPR183, CXCL16 | 121 | 8 | 16792 | 34.69 | 1.0 | 0.53937478 | 59.002592 |
| BP | GO:0034128~negative regulation of MyD88-independent toll-like receptor signaling pathway | 2 | 0.01 | 0.055771 | LY96, CD14 | 121 | 8 | 16792 | 34.69 | 1.0 | 0.53937478 | 59.002592 |
| BP | GO:0071223~cellular response to lipoteichoic acid | 2 | 0.01 | 0.062522 | TREM2, CD14 | 121 | 9 | 16792 | 30.84 | 1.0 | 0.57636345 | 63.327719 |
| BP | GO:0071404~cellular response to low-density lipoprotein particle stimulus | 2 | 0.01 | 0.062522 | FCER1G, ITGB2 | 121 | 9 | 16792 | 30.84 | 1.0 | 0.57636345 | 63.327719 |
| BP | GO:0003094~glomerular filtration | 2 | 0.01 | 0.062522 | JCHAIN, IGHA2 | 121 | 9 | 16792 | 30.84 | 1.0 | 0.57636345 | 63.327719 |
| BP | GO:0019221~cytokine-mediated signaling pathway | 4 | 0.02 | 0.067564 | ASPN, CCL2, IL10RA, HCK | 121 | 131 | 16792 | 4.24 | 1.0 | 0.60017713 | 66.275457 |
| BP | GO:0042102~positive regulation of T cell proliferation | 3 | 0.02 | 0.068565 | CORO1A, TNFSF13B, HLA-DMB | 121 | 60 | 16792 | 6.94 | 1.0 | 0.60032943 | 66.833368 |
| BP | GO:0032691~negative regulation of interleukin-1 beta production | 2 | 0.01 | 0.069225 | ARRB2, ACP5 | 121 | 10 | 16792 | 27.76 | 1.0 | 0.59861972 | 67.196774 |
| BP | GO:0002430~complement receptor mediated signaling pathway | 2 | 0.01 | 0.069225 | C3AR1, FPR3 | 121 | 10 | 16792 | 27.76 | 1.0 | 0.59861972 | 67.196774 |
| BP | GO:0071803~positive regulation of podosome assembly | 2 | 0.01 | 0.069225 | CAPG, LCP1 | 121 | 10 | 16792 | 27.76 | 1.0 | 0.5986197238221174 | 67.19677454371464 |
| BP | GO:0006968~cellular defense response | 3 | 0.02 | 0.072590 | C5AR1, LY96, TYROBP | 121 | 62 | 16792 | 6.72 | 1.0 | 0.61140945 | 68.991613 |
| BP | GO:0050727~regulation of inflammatory response | 3 | 0.02 | 0.074631 | HCK, PYCARD, PIK3AP1 | 121 | 63 | 16792 | 6.61 | 1.0 | 0.61678376 | 70.034942 |
| BP | GO:0016064~immunoglobulin mediated immune response | 2 | 0.01 | 0.075881 | FCER1G, CD74 | 121 | 11 | 16792 | 25.23 | 1.0 | 0.61799461 | 70.657826 |
| BP | GO:0034374~low-density lipoprotein particle remodeling | 2 | 0.01 | 0.075881 | APOE, PLA2G7 | 121 | 11 | 16792 | 25.23 | 1.0 | 0.61799461 | 70.657826 |
| BP | GO:0030574~collagen catabolic process | 3 | 0.02 | 0.076689 | MMP9, CTSS, MMP12 | 121 | 64 | 16792 | 6.51 | 1.0 | 0.61697993 | 71.054263 |
| MF | GO:0042834~peptidoglycan binding | 2 | 0.01 | 0.076798 | JCHAIN, TREM2 | 113 | 12 | 16881 | 24.90 | 0.99999999 | 0.7706674114161207 | 64.76810473040068 |
| BP | GO:0008360~regulation of cell shape | 4 | 0.02 | 0.078952 | CORO1A, CCL2, HCK, ITGB2 | 121 | 140 | 16792 | 3.97 | 1.0 | 0.62311158 | 72.136813 |
| BP | GO:0030595~leukocyte chemotaxis | 2 | 0.01 | 0.082489 | GPR183, CORO1A | 121 | 12 | 16792 | 23.13 | 1.0 | 0.63499427 | 73.753879 |
| BP | GO:0032695~negative regulation of interleukin-12 production | 2 | 0.01 | 0.082489 | ARRB2, ACP5 | 121 | 12 | 16792 | 23.13 | 1.0 | 0.63499427 | 73.753879 |
| BP | GO:0002031~G-protein coupled receptor internalization | 2 | 0.01 | 0.082489 | ARRB2, HTR2B | 121 | 12 | 16792 | 23.13 | 1.0 | 0.63499427 | 73.753879 |
| BP | GO:0006952~defense response | 3 | 0.02 | 0.082972 | ACKR1, TPSAB1, CD74 | 121 | 67 | 16792 | 6.21 | 1.0 | 0.63236599 | 73.967843 |
| BP | GO:0035690~cellular response to drug | 3 | 0.02 | 0.087246 | CCL2, CCL4, PPP1R14A | 121 | 69 | 16792 | 6.03 | 1.0 | 0.64687533 | 75.790254 |
| BP | GO:0002756~MyD88-independent toll-like receptor signaling pathway | 2 | 0.01 | 0.089051 | LY96, CD14 | 121 | 13 | 16792 | 21.35 | 1.0 | 0.65001572 | 76.523407 |
| BP | GO:0034122~negative regulation of toll-like receptor signaling pathway | 2 | 0.01 | 0.089051 | ARRB2, PIK3AP1 | 121 | 13 | 16792 | 21.35 | 1.0 | 0.65001572 | 76.523407 |
| MF | GO:0008201~heparin binding | 4 | 0.02 | 0.090474 | NOV, OGN, CCL2, APOE | 113 | 160 | 16881 | 3.73 | 0.99999999 | 0.80428868 | 71.005528 |
| MF | GO:0032395~MHC class II receptor activity | 2 | 0.01 | 0.095066 | HLA-DMA, HLA-DQA1 | 113 | 15 | 16881 | 19.92 | 0.99999999 | 0.80026828 | 72.859397 |
| CC | GO:0034362~low-density lipoprotein particle | 2 | 0.01 | 0.095399 | APOE, PLA2G7 | 131 | 14 | 18224 | 19.87 | 0.99999997 | 0.463697745 | 70.711707 |
| BP | GO:0042311~vasodilation | 2 | 0.01 | 0.095565 | APOE, ITGA1 | 121 | 14 | 16792 | 19.83 | 1.0 | 0.67252635 | 79.000831 |
| BP | GO:0050965~detection of temperature stimulus involved in sensory perception of pain | 2 | 0.01 | 0.095565 | ARRB2, ANO1 | 121 | 14 | 16792 | 19.83 | 1.0 | 0.67252635 | 79.000831 |
| BP | GO:0010875~positive regulation of cholesterol efflux | 2 | 0.01 | 0.095565 | APOE, PLTP | 121 | 14 | 16792 | 19.83 | 1.0 | 0.67252635 | 79.000831 |
| BP | GO:0030833~regulation of actin filament polymerization | 2 | 0.01 | 0.095565 | PFN2, CORO1A | 121 | 14 | 16792 | 19.83 | 1.0 | 0.67252635 | 79.000831 |
