# Supplementary material for: Identification and validation of four hub genes involved in the plaque deterioration of atherosclerosis
Source: Aging (Albany NY). 2019 Aug 26;11(16):6469–89. doi: 10.18632/aging.102200 (PMC6738408; doi:10.18632/aging.102200)
Supplement: Supplementary Table 2 [file aging-11-102200-s001.docx]

| ##Databases: KEGG PATHWAY |  |  |  |  |  |  |  |
| --- | --- | --- | --- | --- | --- | --- | --- |
| ##Statistical test method: hypergeometric test / Fisher's exact test | | | | |  |  |  |
| ##FDR correction method: Benjamini and Hochberg | | | | |  |  |  |
| **Supplementary Table 2-1. KEGG PATHWAY result of upregulated genes in differentiating plaque sets** | | | | | | | |
| #Term | Database | ID | Input number | Background number | P-Value | Corrected P-Value | Input |
| Rheumatoid arthritis | KEGG PATHWAY | hsa05323 | 4 | 91 | 4.38E-07 | 1.50E-05 | 54\|4312\|414062\|6348 |
| Toll-like receptor signaling pathway | KEGG PATHWAY | hsa04620 | 4 | 106 | 7.89E-07 | 1.50E-05 | 6348\|414062\|6696\|6351 |
| Chemokine signaling pathway | KEGG PATHWAY | hsa04062 | 4 | 187 | 7.08E-06 | 8.97E-05 | 6362\|6348\|414062\|6351 |
| Salmonella infection | KEGG PATHWAY | hsa05132 | 3 | 86 | 2.71E-05 | 0.000207 | 6348\|414062\|6351 |
| Cytokine-cytokine receptor interaction | KEGG PATHWAY | hsa04060 | 4 | 265 | 2.72E-05 | 0.000207 | 6362\|6348\|414062\|6351 |
| Bladder cancer | KEGG PATHWAY | hsa05219 | 2 | 41 | 0.000365 | 0.00231 | 4318\|4312 |
| ECM-receptor interaction | KEGG PATHWAY | hsa04512 | 2 | 82 | 0.001385 | 0.007519 | 6696\|3381 |
| Chagas disease (American trypanosomiasis) | KEGG PATHWAY | hsa05142 | 2 | 104 | 0.002192 | 0.010411 | 6348\|414062 |
| Leukocyte transendothelial migration | KEGG PATHWAY | hsa04670 | 2 | 118 | 0.002796 | 0.011807 | 4318\|1535 |
| Osteoclast differentiation | KEGG PATHWAY | hsa04380 | 2 | 132 | 0.00347 | 0.013187 | 54\|1535 |
| Phenylalanine, tyrosine and tryptophan biosynthesis | KEGG PATHWAY | hsa00400 | 1 | 5 | 0.003915 | 0.013524 | 259307 |
| Focal adhesion | KEGG PATHWAY | hsa04510 | 2 | 203 | 0.007915 | 0.025065 | 6696\|3381 |
| Phenylalanine metabolism | KEGG PATHWAY | hsa00360 | 1 | 17 | 0.0117 | 0.034201 | 259307 |
| MicroRNAs in cancer | KEGG PATHWAY | hsa05206 | 2 | 299 | 0.016449 | 0.044647 | 4318\|3162 |
| PI3K-Akt signaling pathway | KEGG PATHWAY | hsa04151 | 2 | 342 | 0.021129 | 0.052013 | 6696\|3381 |
| Tyrosine metabolism | KEGG PATHWAY | hsa00350 | 1 | 35 | 0.023269 | 0.052013 | 259307 |
| Alanine, aspartate and glutamate metabolism | KEGG PATHWAY | hsa00250 | 1 | 35 | 0.023269 | 0.052013 | 259307 |
| Tryptophan metabolism | KEGG PATHWAY | hsa00380 | 1 | 40 | 0.026459 | 0.052861 | 259307 |
| Porphyrin and chlorophyll metabolism | KEGG PATHWAY | hsa00860 | 1 | 42 | 0.027733 | 0.052861 | 3162 |
| Pathways in cancer | KEGG PATHWAY | hsa05200 | 2 | 397 | 0.027822 | 0.052861 | 4318\|4312 |
| Cysteine and methionine metabolism | KEGG PATHWAY | hsa00270 | 1 | 45 | 0.02964 | 0.053633 | 259307 |
| Valine, leucine and isoleucine degradation | KEGG PATHWAY | hsa00280 | 1 | 48 | 0.031543 | 0.054483 | 259307 |
| Mineral absorption | KEGG PATHWAY | hsa04978 | 1 | 52 | 0.034075 | 0.056298 | 3162 |
| Cytosolic DNA-sensing pathway | KEGG PATHWAY | hsa04623 | 1 | 64 | 0.041634 | 0.06592 | 6351 |
| PPAR signaling pathway | KEGG PATHWAY | hsa03320 | 1 | 72 | 0.046641 | 0.069992 | 4312 |
| Leishmaniasis | KEGG PATHWAY | hsa05140 | 1 | 74 | 0.047889 | 0.069992 | 1535 |
| NF-kappa B signaling pathway | KEGG PATHWAY | hsa04064 | 1 | 93 | 0.059666 | 0.083019 | 6351 |
| Endocrine resistance | KEGG PATHWAY | hsa01522 | 1 | 97 | 0.062128 | 0.083019 | 4318 |
| Estrogen signaling pathway | KEGG PATHWAY | hsa04915 | 1 | 99 | 0.063356 | 0.083019 | 4318 |
| HIF-1 signaling pathway | KEGG PATHWAY | hsa04066 | 1 | 103 | 0.065809 | 0.083358 | 3162 |
| TNF signaling pathway | KEGG PATHWAY | hsa04668 | 1 | 110 | 0.070085 | 0.085911 | 4318 |
| Lysosome | KEGG PATHWAY | hsa04142 | 1 | 123 | 0.077977 | 0.092598 | 54 |
| Wnt signaling pathway | KEGG PATHWAY | hsa04310 | 1 | 143 | 0.089994 | 0.102581 | 4316 |
| Hepatitis B | KEGG PATHWAY | hsa05161 | 1 | 146 | 0.091783 | 0.102581 | 4318 |
| Phagosome | KEGG PATHWAY | hsa04145 | 1 | 155 | 0.097131 | 0.105457 | 1535 |
| Transcriptional misregulation in cancer | KEGG PATHWAY | hsa05202 | 1 | 180 | 0.111828 | 0.118041 | 4318 |
| Proteoglycans in cancer | KEGG PATHWAY | hsa05205 | 1 | 205 | 0.126295 | 0.129708 | 4318 |
| Metabolic pathways | KEGG PATHWAY | hsa01100 | 1 | 1243 | 0.562288 | 0.562288 | 259307 |

| ##Databases: KEGG PATHWAY |  |  |  |  |  |  |  |
| --- | --- | --- | --- | --- | --- | --- | --- |
| ##Statistical test method: hypergeometric test / Fisher's exact test | | | | |  |  |  |
| ##FDR correction method: Benjamini and Hochberg | | | | |  |  |  |
| **Supplementary Table 2-2. KEGG PATHWAY result of downregulated genes in differentiating plaque sets** | | | | | | | |
| #Term | Database | ID | Input number | Background number | P-Value | Corrected P-Value | Input |
| Glycerophospholipid metabolism | KEGG PATHWAY | hsa00564 | 2 | 95 | 0.00 | 0.06 | 139189\|5320 |
| Ras signaling pathway | KEGG PATHWAY | hsa04014 | 2 | 228 | 0.01 | 0.12 | 2246\|5320 |
| alpha-Linolenic acid metabolism | KEGG PATHWAY | hsa00592 | 1 | 25 | 0.02 | 0.12 | 5320 |
| Linoleic acid metabolism | KEGG PATHWAY | hsa00591 | 1 | 29 | 0.02 | 0.12 | 5320 |
| Pathways in cancer | KEGG PATHWAY | hsa05200 | 2 | 397 | 0.02 | 0.12 | 2246\|7185 |
| Fat digestion and absorption | KEGG PATHWAY | hsa04975 | 1 | 41 | 0.03 | 0.12 | 5320 |
| Ether lipid metabolism | KEGG PATHWAY | hsa00565 | 1 | 45 | 0.03 | 0.12 | 5320 |
| Glycerolipid metabolism | KEGG PATHWAY | hsa00561 | 1 | 59 | 0.04 | 0.12 | 139189 |
| Arachidonic acid metabolism | KEGG PATHWAY | hsa00590 | 1 | 62 | 0.04 | 0.12 | 5320 |
| Melanoma | KEGG PATHWAY | hsa05218 | 1 | 71 | 0.04 | 0.12 | 2246 |
| PPAR signaling pathway | KEGG PATHWAY | hsa03320 | 1 | 72 | 0.04 | 0.12 | 729359 |
| Arrhythmogenic right ventricular cardiomyopathy (ARVC) | KEGG PATHWAY | hsa05412 | 1 | 74 | 0.04 | 0.12 | 1674 |
| Hypertrophic cardiomyopathy (HCM) | KEGG PATHWAY | hsa05410 | 1 | 83 | 0.05 | 0.12 | 1674 |
| Small cell lung cancer | KEGG PATHWAY | hsa05222 | 1 | 86 | 0.05 | 0.12 | 7185 |
| Dilated cardiomyopathy | KEGG PATHWAY | hsa05414 | 1 | 90 | 0.05 | 0.12 | 1674 |
| Morphine addiction | KEGG PATHWAY | hsa05032 | 1 | 91 | 0.05 | 0.12 | 8622 |
| NF-kappa B signaling pathway | KEGG PATHWAY | hsa04064 | 1 | 93 | 0.06 | 0.12 | 7185 |
| Pancreatic secretion | KEGG PATHWAY | hsa04972 | 1 | 96 | 0.06 | 0.12 | 5320 |
| Phosphatidylinositol signaling system | KEGG PATHWAY | hsa04070 | 1 | 98 | 0.06 | 0.12 | 139189 |
| Choline metabolism in cancer | KEGG PATHWAY | hsa05231 | 1 | 101 | 0.06 | 0.12 | 139189 |
| TNF signaling pathway | KEGG PATHWAY | hsa04668 | 1 | 110 | 0.06 | 0.13 | 7185 |
| Vascular smooth muscle contraction | KEGG PATHWAY | hsa04270 | 1 | 120 | 0.07 | 0.13 | 5320 |
| Ubiquitin mediated proteolysis | KEGG PATHWAY | hsa04120 | 1 | 137 | 0.08 | 0.13 | 10054 |
| Apoptosis | KEGG PATHWAY | hsa04210 | 1 | 140 | 0.08 | 0.13 | 7185 |
| Wnt signaling pathway | KEGG PATHWAY | hsa04310 | 1 | 143 | 0.08 | 0.13 | 6422 |
| Phospholipase D signaling pathway | KEGG PATHWAY | hsa04072 | 1 | 144 | 0.08 | 0.13 | 139189 |
| Hippo signaling pathway | KEGG PATHWAY | hsa04390 | 1 | 154 | 0.09 | 0.14 | 2246 |
| cGMP-PKG signaling pathway | KEGG PATHWAY | hsa04022 | 1 | 167 | 0.10 | 0.14 | 152 |
| Purine metabolism | KEGG PATHWAY | hsa00230 | 1 | 176 | 0.10 | 0.14 | 8622 |
| Transcriptional misregulation in cancer | KEGG PATHWAY | hsa05202 | 1 | 180 | 0.10 | 0.14 | 7185 |
| Herpes simplex infection | KEGG PATHWAY | hsa05168 | 1 | 186 | 0.11 | 0.14 | 7185 |
| Chemokine signaling pathway | KEGG PATHWAY | hsa04062 | 1 | 187 | 0.11 | 0.14 | 9547 |
| Epstein-Barr virus infection | KEGG PATHWAY | hsa05169 | 1 | 204 | 0.12 | 0.14 | 7185 |
| Viral carcinogenesis | KEGG PATHWAY | hsa05203 | 1 | 205 | 0.12 | 0.14 | 7185 |
| Rap1 signaling pathway | KEGG PATHWAY | hsa04015 | 1 | 211 | 0.12 | 0.14 | 2246 |
| Regulation of actin cytoskeleton | KEGG PATHWAY | hsa04810 | 1 | 215 | 0.12 | 0.14 | 2246 |
| MAPK signaling pathway | KEGG PATHWAY | hsa04010 | 1 | 255 | 0.14 | 0.16 | 2246 |
| Cytokine-cytokine receptor interaction | KEGG PATHWAY | hsa04060 | 1 | 265 | 0.15 | 0.16 | 9547 |
| Neuroactive ligand-receptor interaction | KEGG PATHWAY | hsa04080 | 1 | 278 | 0.16 | 0.16 | 152 |
| Metabolic pathways | KEGG PATHWAY | hsa01100 | 2 | 1243 | 0.17 | 0.18 | 139189\|5320 |
| PI3K-Akt signaling pathway | KEGG PATHWAY | hsa04151 | 1 | 342 | 0.19 | 0.19 | 2246 |
