# Supplementary material for: Identification and validation of four hub genes involved in the plaque deterioration of atherosclerosis
Source: Aging (Albany NY). 2019 Aug 26;11(16):6469–89. doi: 10.18632/aging.102200 (PMC6738408; doi:10.18632/aging.102200)
Supplement: Supplementary Table 3 [file aging-11-102200-s002.docx]

| ##Databases: KEGG PATHWAY | | | | | | |  |
| --- | --- | --- | --- | --- | --- | --- | --- |
| ##Statistical test method: hypergeometric test / Fisher's exact test | | | | | | |  |
| ##FDR correction method: Benjamini and Hochberg | | | | | | |  |
| **Supplementary Table 3. KEGG PATHWAY result of poor prognosis sets** | | | | | | | |
| #Term | Database | ID | Input number | Background number | P-Value | Corrected P-Value | Input |
| Staphylococcus aureus infection | KEGG PATHWAY | hsa05150 | 12 | 57 | 1.12E-17 | 1.31E-15 | 2213\|3689\|3108\|3109\|2215\|3117\|714\|713\|712\|2359\|719\|728 |
| Tuberculosis | KEGG PATHWAY | hsa05152 | 12 | 179 | 3.09E-12 | 1.81E-10 | 2213\|3689\|3108\|3109\|2215\|2207\|3117\|3587\|929\|1520\|11151\|972 |
| Phagosome | KEGG PATHWAY | hsa04145 | 10 | 155 | 3.02E-10 | 1.18E-08 | 2213\|3689\|3108\|3109\|2215\|3117\|4689\|929\|1520\|11151 |
| Complement and coagulation cascades | KEGG PATHWAY | hsa04610 | 8 | 79 | 6.90E-10 | 2.02E-08 | 3689\|719\|714\|713\|712\|11326\|728\|2162 |
| Rheumatoid arthritis | KEGG PATHWAY | hsa05323 | 8 | 91 | 1.95E-09 | 4.57E-08 | 3689\|3108\|3109\|6347\|3117\|54\|10673\|414062 |
| Pertussis | KEGG PATHWAY | hsa05133 | 7 | 75 | 1.41E-08 | 2.74E-07 | 3689\|714\|29108\|23643\|713\|712\|929 |
| Chemokine signaling pathway | KEGG PATHWAY | hsa04062 | 9 | 187 | 2.71E-08 | 4.53E-07 | 6347\|6351\|58191\|7852\|5880\|414062\|6362\|3055\|409 |
| Intestinal immune network for IgA production | KEGG PATHWAY | hsa04672 | 6 | 50 | 3.96E-08 | 5.80E-07 | 3108\|3109\|3117\|608\|7852\|10673 |
| Leishmaniasis | KEGG PATHWAY | hsa05140 | 6 | 74 | 3.43E-07 | 4.46E-06 | 3689\|3108\|3109\|2215\|3117\|4689 |
| Antigen processing and presentation | KEGG PATHWAY | hsa04612 | 6 | 78 | 4.59E-07 | 4.96E-06 | 3108\|3109\|3117\|1520\|10437\|972 |
| Cytokine-cytokine receptor interaction | KEGG PATHWAY | hsa04060 | 9 | 265 | 4.66E-07 | 4.96E-06 | 6351\|6347\|3587\|10673\|58191\|608\|7852\|414062\|6362 |
| Systemic lupus erythematosus | KEGG PATHWAY | hsa05322 | 7 | 136 | 6.51E-07 | 6.34E-06 | 3108\|3109\|2215\|3117\|714\|713\|712 |
| Viral myocarditis | KEGG PATHWAY | hsa05416 | 5 | 60 | 3.00E-06 | 2.70E-05 | 3117\|3689\|5880\|3108\|3109 |
| Asthma | KEGG PATHWAY | hsa05310 | 4 | 32 | 6.99E-06 | 5.84E-05 | 3117\|3108\|3109\|2207 |
| Osteoclast differentiation | KEGG PATHWAY | hsa04380 | 6 | 132 | 8.34E-06 | 6.51E-05 | 2213\|2215\|7305\|54\|54209\|4689 |
| Salmonella infection | KEGG PATHWAY | hsa05132 | 5 | 86 | 1.57E-05 | 0.000115 | 5217\|929\|29108\|414062\|6351 |
| Type I diabetes mellitus | KEGG PATHWAY | hsa04940 | 4 | 44 | 2.24E-05 | 0.000154 | 3117\|3108\|1363\|3109 |
| Chagas disease (American trypanosomiasis) | KEGG PATHWAY | hsa05142 | 5 | 104 | 3.76E-05 | 0.000244 | 714\|713\|712\|6347\|414062 |
| Toll-like receptor signaling pathway | KEGG PATHWAY | hsa04620 | 5 | 106 | 4.10E-05 | 0.000253 | 929\|23643\|6696\|414062\|6351 |
| Leukocyte transendothelial migration | KEGG PATHWAY | hsa04670 | 5 | 118 | 6.69E-05 | 0.000387 | 7852\|4318\|3689\|5880\|4689 |
| Toxoplasmosis | KEGG PATHWAY | hsa05145 | 5 | 119 | 6.95E-05 | 0.000387 | 3117\|23643\|3108\|3109\|3587 |
| Regulation of actin cytoskeleton | KEGG PATHWAY | hsa04810 | 6 | 215 | 0.000117 | 0.00062 | 3689\|8515\|5217\|929\|5880\|3672 |
| Natural killer cell mediated cytotoxicity | KEGG PATHWAY | hsa04650 | 5 | 135 | 0.000123 | 0.000627 | 7305\|3689\|5880\|2215\|2207 |
| B cell receptor signaling pathway | KEGG PATHWAY | hsa04662 | 4 | 73 | 0.000144 | 0.000703 | 2213\|118788\|5880\|971 |
| Prion diseases | KEGG PATHWAY | hsa05020 | 3 | 35 | 0.0003 | 0.001402 | 714\|713\|712 |
| NF-kappa B signaling pathway | KEGG PATHWAY | hsa04064 | 4 | 93 | 0.00035 | 0.001576 | 10673\|929\|23643\|6351 |
| Allograft rejection | KEGG PATHWAY | hsa05330 | 3 | 39 | 0.000404 | 0.001693 | 3117\|3108\|3109 |
| Influenza A | KEGG PATHWAY | hsa05164 | 5 | 176 | 0.000405 | 0.001693 | 3117\|29108\|3108\|3109\|6347 |
| Graft-versus-host disease | KEGG PATHWAY | hsa05332 | 3 | 42 | 0.000495 | 0.001997 | 3117\|3108\|3109 |
| Herpes simplex infection | KEGG PATHWAY | hsa05168 | 5 | 186 | 0.000517 | 0.002018 | 3117\|3108\|3109\|6347\|972 |
| Malaria | KEGG PATHWAY | hsa05144 | 3 | 49 | 0.000758 | 0.00286 | 2532\|3689\|6347 |
| Autoimmune thyroid disease | KEGG PATHWAY | hsa05320 | 3 | 54 | 0.000991 | 0.003623 | 3117\|3108\|3109 |
| Legionellosis | KEGG PATHWAY | hsa05134 | 3 | 55 | 0.001042 | 0.003696 | 929\|3689\|29108 |
| Inflammatory bowel disease (IBD) | KEGG PATHWAY | hsa05321 | 3 | 66 | 0.001722 | 0.005927 | 3117\|3108\|3109 |
| Cell adhesion molecules (CAMs) | KEGG PATHWAY | hsa04514 | 4 | 146 | 0.001782 | 0.005957 | 3117\|3689\|3108\|3109 |
| Neuroactive ligand-receptor interaction | KEGG PATHWAY | hsa04080 | 5 | 278 | 0.002932 | 0.009529 | 3357\|2359\|719\|140\|728 |
| ECM-receptor interaction | KEGG PATHWAY | hsa04512 | 3 | 82 | 0.003121 | 0.009868 | 3672\|8515\|6696 |
| Dilated cardiomyopathy | KEGG PATHWAY | hsa05414 | 3 | 90 | 0.00402 | 0.012376 | 3672\|8515\|5350 |
| Fc gamma R-mediated phagocytosis | KEGG PATHWAY | hsa04666 | 3 | 93 | 0.004393 | 0.013179 | 2213\|5880\|3055 |
| Focal adhesion | KEGG PATHWAY | hsa04510 | 4 | 203 | 0.005633 | 0.016477 | 3672\|5880\|6696\|8515 |
| Sphingolipid signaling pathway | KEGG PATHWAY | hsa04071 | 3 | 121 | 0.0089 | 0.025398 | 5880\|140\|2207 |
| Lysosome | KEGG PATHWAY | hsa04142 | 3 | 123 | 0.009296 | 0.025895 | 54\|1520\|7805 |
| HTLV-I infection | KEGG PATHWAY | hsa05166 | 4 | 259 | 0.012776 | 0.034762 | 3117\|3689\|3108\|3109 |
| Pathogenic Escherichia coli infection | KEGG PATHWAY | hsa05130 | 2 | 55 | 0.016289 | 0.043314 | 929\|23643 |
| NOD-like receptor signaling pathway | KEGG PATHWAY | hsa04621 | 2 | 57 | 0.017386 | 0.045203 | 29108\|6347 |
